# Roche Digital Pathology Dx whole slide imaging system is comparable to traditional microscopy for primary diagnosis in surgical pathology

**DOI:** 10.1093/ajcp/aqaf052

**Published:** 2025-06-10

**Authors:** Keith A Wharton, Jim Ranger-Moore, Hon Seng, Alexander D Borowsky, Cynthia A Behling, Nicolas Cacciabeve, Michael LaFriniere, Richard M Feddersen, Crystal Williams, Drew Baldwin, Richard Louie, Lauren Murata, Cameron Smith, Andrea Visoski, Mingfei Zhao, Shalini Singh, Tracie N Gardner

**Affiliations:** Roche Diagnostics Solutions, Oro Valley, AZ, United States; Roche Diagnostics Solutions, Oro Valley, AZ, United States; Roche Diagnostics Solutions, Oro Valley, AZ, United States; Department of Pathology, University of California Davis, Davis, CA, United States; Pacific Rim Pathology, San Diego, CA, United States; Advanced Pathology Associates, Rockville, MD, United States; Advanced Pathology Associates, Rockville, MD, United States; Tricore Reference Laboratories, Albuquerque, NM, United States; Roche Diagnostics Solutions, Oro Valley, AZ, United States; Roche Diagnostics Solutions, Oro Valley, AZ, United States; Roche Diagnostics Solutions, Oro Valley, AZ, United States; Roche Diagnostics Solutions, Oro Valley, AZ, United States; Roche Diagnostics Solutions, Oro Valley, AZ, United States; Roche Diagnostics Solutions, Oro Valley, AZ, United States; Roche Diagnostics Solutions, Oro Valley, AZ, United States; Roche Diagnostics Solutions, Oro Valley, AZ, United States; Roche Diagnostics Solutions, Oro Valley, AZ, United States

**Keywords:** diagnostics, digital pathology, microscopy, primary diagnosis, whole slide imaging

## Abstract

**Objective:**

We evaluated the clinical performance of Roche Digital Pathology Dx, a whole slide imaging (WSI) system, in 2 studies according to US Food and Drug Administration (FDA) and Digital Pathology Association criteria.

**Methods:**

Precision was measured by pathologists identifying 23 histopathology features; accuracy was assessed by comparing diagnoses from 2047 clinical cases with those from manual microscopy, with exploratory analyses including subgroup-specific diagnostic discrepancy rates.

**Results:**

Both studies met all predetermined primary endpoints. Precision between systems/sites was 89.3%; between days, 90.3%; and between readers, 90.1% (lower bound of 95% CI for each, ≥85%). The difference in accuracy between digital reads (DRs) and manual microscopy reads (MRs) vs reference sign-out diagnosis (SD), DRs – MRs, was –0.61% (lower bound of 95% CI, –1.59%), which was greater than the lower bound acceptance criterion (–4%). Mean case reading times were similar: 2.33 minutes (DRs) and 2.34 minutes (MRs). Review of breast, lung, bladder, kidney, and stomach case diagnoses did not identify DR modality-specific root causes for major diagnostic disagreements. Higher than expected disagreements in both modalities were traced to COVID-19 pandemic-related resource constraints, leading to challenging case adjudications and higher disagreement rates for longer SDs. Direct DR/MR adjudication supported this hypothesis, resulting in an intermodality disagreement rate of 4.77%; using SD as a “tiebreaker” reduced the overall DR disagreement rate to 2.97%.

**Conclusions:**

Roche Digital Pathology Dx is noninferior to manual microscopy for primary diagnosis in surgical pathology, with performance results similar to 5 distinct FDA-cleared WSI systems using different scanners.

**KEY POINTS**

- Roche Digital Pathology Dx is noninferior to manual microscopy for surgical pathology primary diagnoses, increasing confidence in whole slide imaging (WSI) for routine diagnosis.
- Performance results align with US Food and Drug Administration 510(k) clearance studies of 5 WSI systems; no digital modality-specific causes for breast, lung, bladder, kidney, and stomach diagnostic disagreements were found.
- Higher digital and manual microscopy diagnostic discrepancy rates vs prior WSI system studies were due to difficulty adjudicating long-reference sign-out diagnoses with shorter reader-generated ones.

## INTRODUCTION

Since the 19th century, medical practice has relied on pathologists to establish a patient’s primary diagnosis through microscopic examination of formalin-fixed, paraffin-embedded tissue sections stained with hematoxylin and eosin (H&E), and, increasingly, with ancillary histochemical (“special”) stains and molecular labeling assays such as immunohistochemistry (IHC) and in situ hybridization (ISH). Primary diagnosis informs the standard of care, prognosis, biomarker testing, therapy selection, monitoring for disease surveillance, and clinical trial eligibility, and it remains an organizing principle for investigations. Whole slide imaging (WSI), which uses slide scanners and dynamic, data pyramid–based image rendering, re-creates the tissue-viewing experience of a microscope on a computer display.^1,2^ WSI is increasingly favored over traditional microscopy for education and research in academic and industrial settings,^3,4^ has been validated for diagnostic use in a wide variety of animal and human contexts,^5-17^ and will be instrumental for wider use of computational pathology and artificial intelligence (AI) in pathology practice.^18,19^

Recognition of features, clinical context, and language are building blocks of primary diagnosis. Histologic features include diverse cell types and normal and abnormal structures seen in various pathologic lesions and diseases. AI investigations have identified thousands of human interpretable features (HIFs) as the basis of deep learning models, providing a measure of explainability for “black-box” AI algorithm outputs.^20^ In cancer, some HIFs correspond to features of the tumor-immune microenvironment, such as lymphocyte density, tumor necrosis, or fibroblast architecture.^21^ Clinical context includes the patient’s history, surgical procedure, and gross description of lesions sampled for pathology. When correlated with clinical and gross specimen observations, each diagnosis relies on the identification of some features and the absence of others. The words and phrases used to describe lesions and their features are continually evolving due to advances in understanding disease, underscoring the need for a shared contemporary vocabulary. Yet, misnomers persist: the “pyogenic granuloma” remains neither pyogenic nor granulomatous. AI-based large language models trained with pathology data aim to improve pathologists’ access to knowledge and diagnostic support, but a myriad of challenges to adoption remain.^22^ Thus, these 3 building blocks are essential when investigating WSI for primary diagnosis.

WSI systems consist of 3 components: (1) slide scanner, (2) viewing software, and (3) display monitor.^23^ The pixel “pathway” or “pipeline” describes information transit through the system, from glass slide to WSI viewed on a monitor.^24^ US Food and Drug Administration (FDA) 510(k)-cleared WSI systems have similar designs but distinct pixel pathways and noninterchangeable components. Despite increased awareness of WSI’s utility for primary diagnosis, acutely recognized at the onset of the COVID-19 pandemic, WSI adoption has lagged, particularly in the United States,^25-27^ in part due to a limited number of cleared systems being available since the first clearance in 2017.^24,28-32^ All FDA-cleared systems with unique scanners have passed 2 types of rigorous clinical validation studies, the scopes of which were originally negotiated between the FDA, the Digital Pathology Association (DPA), and various manufacturers.^24,33-36^

Here we describe Roche Digital Pathology Dx and the results of an interlaboratory reproducibility (ILR)/precision study of 23 features and a method comparison (MC)/accuracy study of diagnoses from 2047 cases rendered by pathologists compared with manual microscopy. Both studies achieved all predefined acceptance criteria, serving as a basis for FDA 510(k) clearances on June 14, 2024^37^ (K232879), and December 16, 2024 (K242783).^37,38^ Our results build confidence in digital pathology, specifically in the Roche Digital Pathology Dx system, for primary diagnosis in surgical pathology.

## METHODS

Roche Digital Pathology Dx (hereafter referred to as “RDPD,” or the “System”) is an automated digital creation, viewing, and management system established with reference to guidance^23^ from the following components:

1. *Slide Scanner*: VENTANA DP 200 scanner (Ventana Medical Systems, Inc., Roche Diagnostics International Ltd, and F. Hoffmann-La Roche Ltd), which was used in the 2 clinical performance studies, is a 6-slide single-tray slide scanner that uses a 20× objective lens and stripe scanning format to capture sharp, 20× or 40× digital WSIs in proprietary BIF format (used on System), or widely available TIF or DICOM formats. Available globally since 2018, VENTANA DP 200 scanner features include automated 1-dimensional and 2-dimensional barcode reading and tissue detection, consistent color profiles from International Color Consortium profile management, and the ability to scan up to 15 z-axis layers. The VENTANA DP 600 scanner, available globally since 2022, is a high capacity, 240-slide multiple tray scanner with an image acquisition unit (including optical and electronic pixel pipeline) that is identical by design to the VENTANA DP 200 scanner; it was added to the RDPD device clearance through FDA’s Special 510(k) pathway (K242783).^38^
2. *Software*: Roche uPath enterprise software is a local server-based WSI management and workflow software application, accessed via web browser, with acquisition, management, viewing, annotation, analysis, image sharing, and reporting capabilities. Version 1.1 software was loaded on local, dedicated internet-connected computer workstations running Windows 10. Images were retrieved from onsite servers and viewed on the Google Chrome web browser (Note: “uPath” is currently “navify Digital Pathology”).
3. *Monitor*: ASUS ProArt Display PA248QV (ASUSTek Computer, Inc., Taipei, Taiwan) is a commercially available professional display monitor that meets or exceeds specifications of viewing monitors previously cleared as part of WSI systems.

Usability testing confirmed that RDPD allowed completion of all critical tasks required for primary diagnosis (data not shown). Study sites, including academic and nonacademic clinical laboratories, are listed in **Table S1**. Study protocols were approved by study site institutional review boards. Systems were affixed with labels indicating they were for “Investigational Use Only” as candidates for FDA 510(k) clearance. Systems were qualified and calibrated as properly functioning by dedicated study personnel following site installation, then returned to the sponsor at study completion.

**Figure 1:**
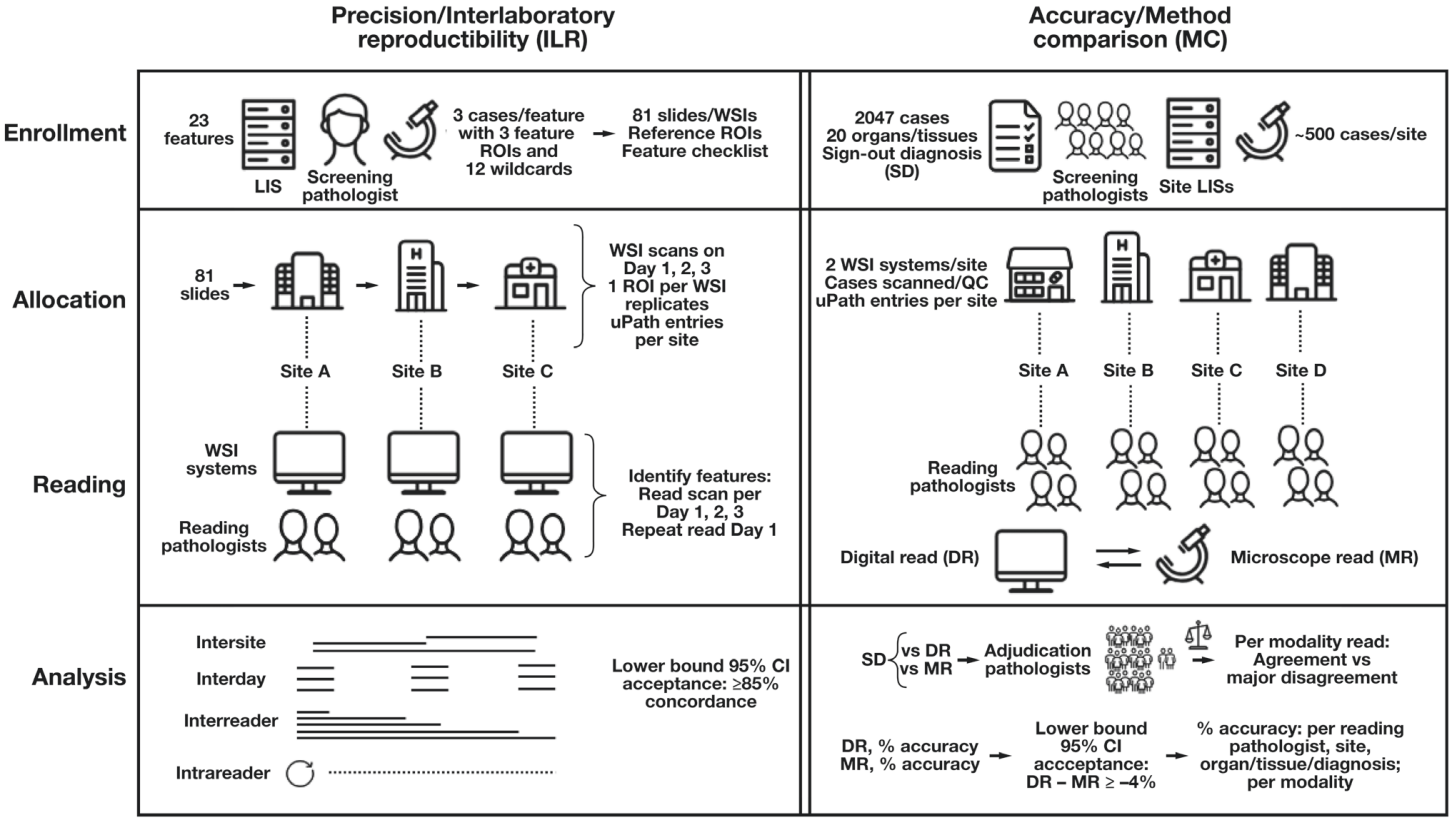
Clinical performance study designs. Precision/interlaboratory reproducibility (left) and accuracy/method comparison (right) study workflows, consisting of enrollment, allocation, reading, and analysis stages. See Methods for details. DR, digital read; ILR, interlaboratory reproducibility; LIS, laboratory information system; MC, method comparison; MR, manual microscopy read; QC, quality control; ROI, region of interest; SD, sign-out diagnosis; WSI, whole slide imaging.

Study designs were based on guidelines suggested by the FDA and DPA and similar to those of cleared predicates **Figure 1**.^24,33-36^ Pathologist roles for both studies comprised screening, reading, and adjudication, with 1 role allowed per pathologist per study; all pathologists were licensed to practice medicine, were board-certified in pathology, and had experience practicing surgical pathology (see Acknowledgments).

### Precision/ILR study

This study tested repeatability and reproducibility (precision) of RDPD for the identification of 23 histopathologic features across 3 sites (**Figure 1**, Table S1). A single screening pathologist identified 3 different slides with distinct sample types or diseases containing at least 3 examples of 1 of the 23 features, visualized either by 20× (n = 12 features) or 40× (n = 11 features) magnification (Table S2), resulting in enrollment of 23 × 3 (= 69) glass slide “cases.” There were 3 regions of interest (ROIs), with each ROI including at least 1 example of that slide’s primary feature, were selected from each case. The ROIs may have had additional incidental features present on the feature list; these additional features, if present, were neither recorded by the screening pathologist nor included in statistical analysis. In addition, 12 “wildcard” cases with 3 ROIs, each ROI including at least 1 listed primary feature, for a total of 36 ROIs, were randomized in each reading workflow. Thus, a set of 69 + 12 (= 81) cases (slides) was assembled. This slide set was scanned on 3 nonconsecutive days on the single system at each site, for a total of 3 × 81 (= 243) WSIs at each site and a total of 3 × 243 (= 729) WSIs for all sites, with each of the 3 × 729 (= 2187) ROIs delimited by a green box on each WSI. Although 3 ROIs with the same primary feature were selected from each case slide used in the study (ie, excluding wildcards, each slide may have included more than 1 primary feature), only 1 ROI was indicated on each displayed WSI at a time so the reader would not use other features/ROIs from the same WSI to more easily identify the correct feature. There were 6 reading pathologists (2 per site) who assessed ROIs and were asked to select the feature(s) present in each ROI on the case report form (CRF) feature checklist in the clinical database. Only features designated as the primary feature by the screening pathologist in each ROI were counted as agreements; readers were neither penalized for incorrect identification of features if the primary feature was correctly identified, nor were they given credit for identification of a feature that, if present in the ROI, was not designated as the primary feature for that ROI. Bias was minimized by: (1) randomized placement of the primary feature within each ROI (so that the feature was not always in the center of the ROI), (2) randomization of source WSI and ROI reading orders, (3) inclusion of wildcard cases excluded from statistical analysis, and (4) 14-day or longer washout period between each reading session. After the first 3 reading sessions, using image sets scanned on each of the 3 scanning days, a fourth reading session reusing the image set from the first scanning day allowed assessment of intrareader variation.

### Accuracy/MC study

This study compared diagnoses from 2047 cases across 4 sites by microscopy (manual read [MR]) or digitally (digital read [DR]) using RDPD with each case’s original sign-out (reference) diagnosis (SD) **Figure 1**. Each site’s screening pathologists assessed more than 500 archival cases from a prespecified target list of diverse organs/tissues and sample types/procedures (eg, tumor resections, biopsies) (Table S3), recording each case’s SD. Enrolled cases, 1 per patient, consisted of H&E tissue section(s) and any ancillary stains required to render a primary diagnosis matching the SD. A minimum of 10% “difficult” case diagnoses was targeted (Table S4).^34^ Slides from each site were verified, deidentified, scanned at 20× on 1 of the 2 systems at that site, and then quality checked; pathologists could request a 40× scan if needed. Each of the 4 reading pathologists (readers) per site was assigned to read all ~500 cases from their site in 4 reading blocks of ~125 cases per block. For each case, the reading pathologist assessed WSI adequacy then established diagnoses by MR and DR, separated by washout periods, and recorded them in the CRF. Next, 2 adjudication pathologists (adjudicators) separately compared each case’s SD with the reader’s MR and DR diagnoses (blinded as to modality and separated by washout periods) and determined whether each pair of diagnoses was concordant (“agree”), a minor discrepancy (“disagree with minor differences,” defined as 2 different diagnoses leading to no clinically significant difference in patient management), a major discrepancy (“disagree with major differences,” defined as 2 different diagnoses leading to a clinically significant difference in patient management), or deferral, if for any reason they could not adjudicate the diagnoses. If the 2 adjudicators disagreed as to whether a discrepancy was major or nonmajor (ie, concordant or a minor discrepancy), or if 1 of them deferred, a third adjudicator served as tiebreaker. If all 3 adjudicators disagreed with each other, an in-person or virtual consensus meeting was conducted for final case declaration. Adjudicators were provided with a diagnosis lookup table that specified whether several indicated pairs of diagnoses were minor vs major discrepancies, similar to that used in the predicate study; however, the lookup table did not include every possible diagnosis, and each final case read determination was at the discretion of each adjudicator.^34^ The study had 20 initial adjudicators: 18 were organized as 13 fixed pairs based on availability (ie, not random) to separately compare DR and MR diagnoses (blinded as to modality) to the SD, with each adjudicator able to serve on more than 1 team and each team assigned to all paired DR and MR diagnoses for each case; the remaining 2 adjudicators served as tiebreakers, with each one managing all third adjudications (if needed) for 2 sites. Of the 20 initial adjudicators, 2 were Roche employees who reviewed 6.8% (515/7562) of all paired case reads; the 2 site tiebreakers were not Roche employees. Direct MR/DR readjudication was performed by 2 Roche employee coauthors (K.A.W. and T.N.G.) as described in the Results, with final consensus derived from a virtual meeting. Bias was minimized by: (1) screening of consecutive cases greater than 1 year old for enrollment, (2) randomization of cases, blocks of cases, and modality reading orders, including a more than 30-day washout period separating DR and MR reads, or vice versa, and (3) multiple pairs of adjudicators blinded to modality, other diagnoses, and other adjudications. Case enrollment and study subpopulations are shown in Figure S1.

### Data collection and statistical analysis

For both ILR and MC studies, 2-sided 95% CIs were calculated from point estimates using the percentile bootstrap method from 2000 samples.

- For the ILR study, overall percent agreement (OPA) of feature identification for all possible pairwise comparisons was calculated between sites/systems, days, and readers. Acceptance criteria for each endpoint required that the lower bound of the 95% CI of the OPA be 85% or more. Intrareader agreement across all pairwise comparisons was calculated without a predefined acceptance criterion.
- The MC study was designed to have more than 99% power to demonstrate noninferiority of DR compared with MR with a margin of –4%, assuming 96% modality accuracy. The difference in accuracy of each modality read (DR – MR) compared to the SD was calculated with 95% CIs, and by reader, site, organ, sample type, and adjudicator team. In addition, a generalized linear mixed-model logistic regression was conducted on the “intent-to-adjudicate” (ITA) population (Figure S1). For each reading modality and result, the dependent variable was the agreement with SD status. The model accounted for fixed study effects (ie, reading modality and organ type) and random study effects (ie, site and readers nested within sites). The overall acceptance criterion required the lower bound of the 95% CI of DR – MR, percent accuracy to be –4% or more, with no predefined acceptance criteria for modality accuracy/discrepancy or subgroup assessments.

All statistical analyses were conducted using SAS software, version 9.4 (SAS Institute), in addition to standard methods for descriptive statistics (cross-tabulation reporting frequencies and percentages, with Wilson score CIs reported). Generalized linear mixed regression analyses were performed using GLIMMIX. Additional logistic regression analyses to investigate the effects of SD length are as described. Data exploration was performed using Google Sheets and Microsoft Excel, and graphs were generated using Prism (GraphPad Software).

The MC study case read times exceeding 30 minutes by minute time stamp (which were less than 1% of reads in either modality [data not shown]) were excluded from mean reading time calculations, assuming the pathologist interrupted their work and later returned without logging out of the system.^34^

#### Study execution and monitoring

In the ILR study, “None of the above” was unintentionally listed as a feature choice for Day 1 reads at all 3 sites and for Day 2 reads at site C. Since all ROIs had 1 or more features (ie, no study or wildcard ROIs had zero listed features), and predicate device studies did not allow such choice, we hypothesized that its inclusion might underestimate true precision. Accordingly, this option was removed from the feature list CRF for the remainder of the study. At study completion, “None of the above” was selected in 1.9% (70/3656) of total study ROI reads; in the primary analysis, all of these reads were counted as a failure to identify the feature, but in a sensitivity analysis, these 70 ROI reads were excluded from calculations.

## RESULTS

### System performance

In the ILR/precision study, readers considered 98.8% (4910/4968) of ROI reads as adequate for evaluation; among the 58 ROI reads specified as inadequate, 46 (79%) of the reads were from WSIs of 5 decalcified bone-containing sections with some areas not mounted flat on slides that readers listed as “image blurred or out of focus”. For the MC study, 3259 slides were scanned: 76.1% (2479) were H&E and 23.9% (780) were ancillary (IHC, ISH, or special histochemical) stains. The number of H&E slides per case ranged from 1 (1761 cases) to 8 (1 case); for cases with ancillary slides, the range was 1 (217 cases) to 10 (5 cases). Initial WSIs were considered acceptable for diagnosis in 96.8% (2400/2479) of H&E slides and 93.6% (730/780) of ancillary slides, with only 2 H&E and 2 ancillary slides unable to generate an acceptable WSI after up to 2 additional rescans—an overall acceptable scanning rate of 99.9%. Consistent with WSIs at 20× magnification being adequate for most diagnoses,^34^ readers requested 40× WSIs for only 6 slides (5 H&E, 1 ancillary) from 4 different cases. Mean case reading time was 2.33 minutes for DR (range, 1.56-3.46 minutes) and 2.34 minutes for MR (range, 1.69-3.14 minutes), a negligible difference.

### ILR/precision study

**Figure 2:**
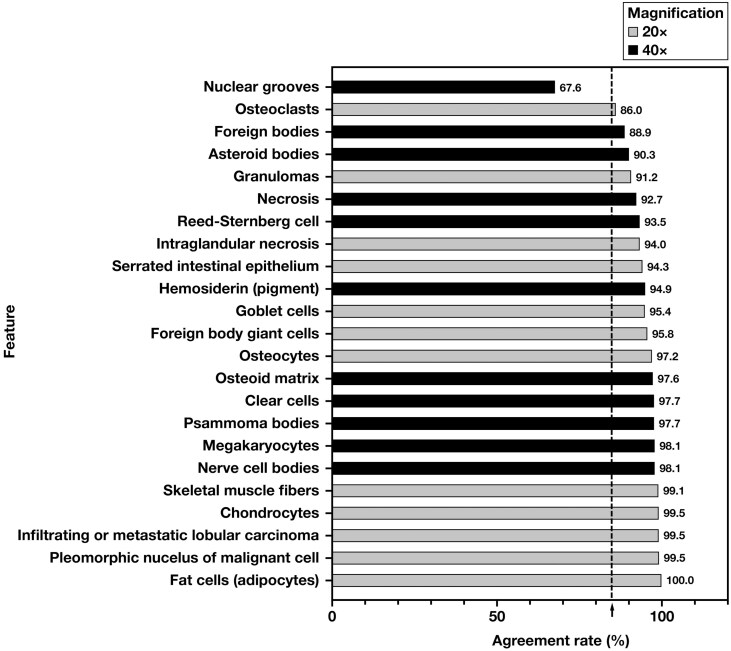
Feature precision. The 23 study features ranked by overall agreement rates, from low to high, with feature magnification indicated by bar color (20×, gray; 40×, black). The vertical dashed line indicates an overall study acceptance criterion of 85%.

The study met all predetermined primary endpoints: intersite/system, interday, and interreader precision were 89.3%, 90.3%, and 90.1%, with all lower limits of the 95% CIs 85% or more **Table 1**. Intrareader precision was 88.1%, ranging from 80.0% to 97.6% among the 6 readers; a sensitivity analysis in which exclusion of 1.9% of total study reads where readers selected “none of the above” features were present in the ROI (see Methods) increased overall intrareader precision to 92.9% (reader range, 89.0%-98.1%) (data not shown). Primary features were correctly identified in 94.3% (4630/4910) of ROI reads. Precision by feature ranged from 100% for “fat cells (adipocytes)” to 67.6% for “nuclear grooves,” with all remaining features except “osteoclasts” and “foreign bodies” with 90% or more precision **Figure 2**.

**Table 1:** Overall Percent Agreement Rates for Precision/Interlaboratory Reproducibility Study—Primary Endpoints

Endpoint	Overall percent agreement (n/N)^a^	95% CI^b^
Intersite/system	89.3 (19 510/21 839)	85.8-92.4
Interday (within-system)	90.3 (3302/3656)	87.1-93.2
Interreader	90.1 (1650/1832)	86.6-93.0

^a^n = number of pairwise comparisons for which the reference primary feature was identified in both assessments. N = total number of pairwise comparisons.

^b^Two-sided 95% CIs were constructed using the percentile bootstrap method from 2000 replicates.

#### Root causes of feature misidentification

**Figure 3:**
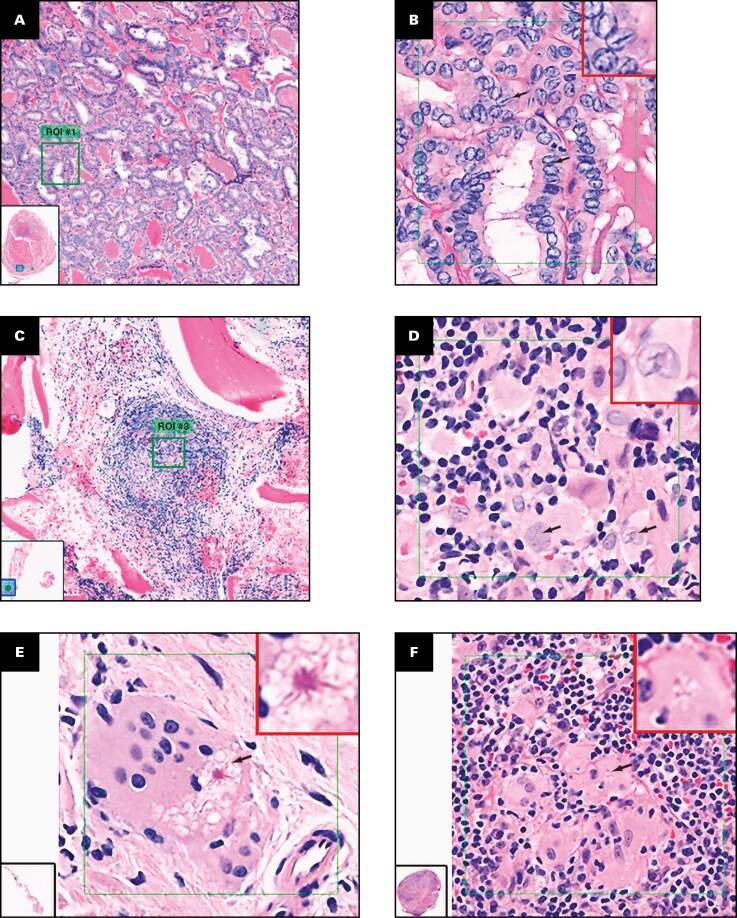
Feature precision examples. **A**, **B**, Papillary carcinoma of the thyroid (case S44). **A**, Scanning magnification and inset WSI thumbnail image showing ROI (green box). **B**, A 40× ROI with green box as displayed in the study viewer window. Arrows indicate abundant nuclear grooves recognized in 100% (24/24) of ROI reads, with a higher-magnification image in the red box in the upper right corner. **C**, **D**, Acetabular bone marrow biopsy specimen with a focus of Langerhans cell histiocytosis (case S45). **C**, Scanning magnification and inset WSI thumbnail image showing ROI (green box). **D**, A 40× ROI with green box as displayed in the study viewer window. Nuclear grooves (arrows and higher-magnification example in red box) within enlarged histiocytic nuclei were recognized in only 4.2% (1/24) of reads of this ROI. **E**, Endobronchial lung biopsy specimen with granulomatous inflammation (case S33), with a lower left WSI thumbnail image. Arrow indicates a classic-appearing asteroid body, with an eosinophilic center and radiating arms, which was identified in 100% of 24 ROI reads. **F**, Lymph node (case S82) with WSI thumbnail image. Arrow shows single minute asteroid body with a small center and inconspicuous arms, yet within a multinucleated giant cell, which was identified in 79% (19/24) of ROI reads. ROI, region of interest; WSI, whole slide imaging.

Case diagnosis, specific feature attributes, and reader identification of all ROI features were important for correct feature identification, illustrated as follows:

- “Nuclear grooves” are curvilinear nuclear indentations seen in neoplasia.^39^ Of the 3 cases, 2 were carcinomas (thyroid, ovary); all 3 ROIs in each were correctly identified in 100% (144/144) of ROI reads **Figure 3A, B**. The third case was a microscopic focus of Langerhans cell histiocytosis (LCH) in bone marrow, characterized by atypical, enlarged histiocyte-like cells admixed with a benign mononuclear infiltrate **Figure 3C, D**. While “nuclear grooves” are a known feature of LCH and were present in each LCH case ROI, they were only identified in 2.8% (2/72) of ROI reads, accounting for the feature’s ranking with the lowest agreement. Among incorrect ROI reads for the LCH case, features most commonly selected were “Reed-Sternberg cells” in 74% (53/72) of ROI reads and “asteroid bodies” in 8.3% (6/72) of ROI reads. These data suggest readers misidentified LCH ROIs as either Hodgkin disease or a granulomatous process and then chose a plausible (but incorrect) listed feature.
- “Reed-Sternberg cells” with atypical features, unlike those with classic “owl’s eyes” features in Hodgkin disease, were among the most challenging features to identify in a predicate device study.^35^ Reed-Sternberg cells in this study, by contrast, had mostly canonical features and were identified in 93.5% (202/216) of ROI reads **Figure 2**.
- “Asteroid bodies”: Among the 3 cases/9 ROIs, correct identification was associated with the feature’s star-like shape **Figure 3E, F**.
- “Osteoclasts” and “osteocytes”: In ROIs with “osteoclasts” as the primary feature (86.0% precision), “osteocytes” were selected as the primary feature in 83% (24/29) of incorrect ROI reads. Conversely, in ROIs with “osteocytes” as the primary feature (97.2% precision), “osteoclasts” were selected as the primary feature in 50% (3/6) of incorrect ROI reads.
- “Foreign body giant cells” and “granulomas”: In one-third of cases with “foreign body giant cells” as the correct feature, 100% of the 9 incorrect (out of 72 total) ROI reads were identified as “granulomas.” Conversely, in one-third of cases with “granulomas” as the correct feature, “foreign body giant cells” was chosen in 100% of the 19 incorrect (out of 72 total) ROI reads.

Because “osteoclasts” are in close proximity to “osteocytes” in bony tissues, and “granulomas” and “foreign body giant cells” often coexist in granulomatous inflammation, we infer that in a subset of ROIs at the same magnification that contained 2 or more features commonly associated with the same tissue/organ or pathologic process, readers sometimes identified a single feature present in the ROI, but the feature they selected for that ROI was not the feature chosen by the screening pathologist for study enrollment.

### MC/accuracy study

Among the 2047 enrolled cases (EC) population, 2032 cases (99.3%; the “all evaluable cases” [AEC] population), consisting of 7562 paired diagnoses (ie, “case reads”) adjudicated in both modalities (DRs and MRs), were analyzed; 15 cases were excluded due to reader case deferral or adjudication failure (Figure S1). Overall, 92.4% (15 124/16 376 case reads) of the total theoretical possible case reads (of the EC population) were fully adjudicated; most cases that were unable to be diagnosed or adjudicated were due to a reader requirement for additional information, need for subspecialty consultation, or for multiple reasons (data not shown).

The study met all prespecified primary endpoints. In the AEC population, accuracy across all study sites was 92.61% (discrepancy 7.39%) for MR and 92.00% (discrepancy 8.00%) for DR, with DR – MR percent accuracy of –0.61% (lower bound of 95% CI, –1.59%) **Table 2**. A sensitivity analysis on the “ITA” cohort of 2037 cases resulted in a MR accuracy of 92.16%, a DR accuracy of 91.54%, and DR – MR percent accuracy of –0.62% **Table 2**. With the lower bound of the 95% CI of study DR – MR percent accuracy being –4% or more, RDPD is demonstrated to be noninferior to manual microscopy for primary diagnosis in surgical pathology.

**Table 2:** Modality Discrepancy Rates for Accuracy/Method Comparison Study—Primary Endpoints

	Digital WSI read (DR)				Manual microscopic read (MR)				DR – MR, % accuracy		
	No. of discrepancies	Total case reads	Major discrepancy rate, %	95% CI	No. of discrepancies	Total case reads	Major discrepancy rate, %	95% CI	No. of discrepancies	%	95% CI
Observed	605	7562	8.00	6.73 to 9.27	559	7562	7.39	6.11 to 8.78	–46	–0.61	–1.59 to 0.35
Model	—	7725	8.46	7.35 to 9.71	—	7744	7.84	6.80 to 9.12	—	–0.62	–1.50 to 0.26

“Observed” is based on study data from the “all evaluable cases” cohort; “Model” is a generalized linear mixed model (GLIMMIX) using the “intent-to-adjudicate” cohort. Abbreviations: DR, digital read; MR, manual microscopic read; WSI, whole slide imaging.

### Intersite and interreader/intrareader

**Figure 4:**
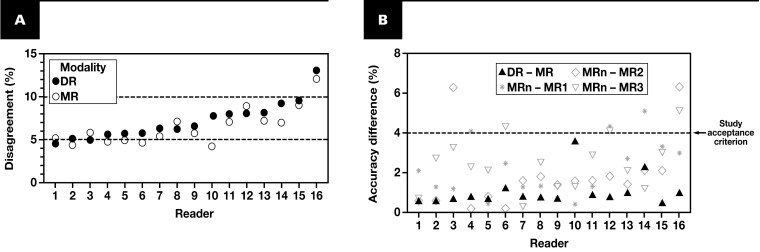
Diagnostic accuracy by modality and reader. **A**, Modality diagnostic major disagreement rate by reader (readers deidentified without regard to site as Nos. 1-16, indicated below bottom graph), ranked from lowest to highest percentage disagreement for their site’s ~500 cases by DR (solid circles), plotted with their percentage major disagreement by MR (open circles). **B**, The absolute value of each reader’s DR – MR percent accuracy (black triangles) compared to the difference between their MR modality read accuracy and that of each of the other 3 readers on the same set of cases at their site, arbitrarily numbered as MR1, MR2, and MR3 (gray symbols) and ranked by reader DR disagreement as in **A**. The absolute value of the study acceptance criterion for DR – MR percent accuracy (4%) is indicated by a dashed line to facilitate comparison with interreader MR accuracy differences by site. DR, digital read; MR, manual microscopy read.

Using the “all completely evaluable cases” subpopulation of 1592 cases (ie, cases where all 4 readers rendered paired MR and DR diagnoses that were both successfully adjudicated, Figure S1), overall intersite DR – MR percent accuracy was –0.75%, ranging from –0.13% to –1.47% by site (data not shown). Interreader DR – MR percent accuracy across all sites was –0.80%, with an overall intrareader MR/DR agreement of 93.9% (data not shown). In the EC population, most readers’ disagreement rates were ~5% to 10%, and for 75% (12/16) of readers, DR disagreement was slightly higher than MR disagreement **Figure 4A**. Notably, DR – MR percent accuracy was –4% or more for 100% (16/16) of readers and typically less than interreader MR accuracy at each site **Figure 4B**. These data indicate that reading modality was a minor contributor to reader and site variation.

### Organ/sample type

Among the 20 organ groups, gallbladder and hernial/peritoneal had the highest modality accuracy at 100%; bladder, gynecologic, and skin had modality accuracies in the 85% to 90% range; and all remaining organs had 1 or both modality accuracies more than 90% **Table 3**.

**Table 3:** Modality Diagnostic Accuracies by Organ Group

Organ	Digital read (DR), % accuracy	Manual read (MR), % accuracy	(DR – MR), % accuracy
Anus/perianal	93.0	95.7	–2.7
Appendix	98.4	100.0	–1.6
Bladder	85.9	87.8	–1.8
Brain/neurologic	94.3	92.4	1.9
Breast	91.0	93.2	–2.1
Colorectal	93.2	93.0	0.2
Endocrine	91.3	92.1	–0.8
Gastroesophageal junction	90.7	91.5	–0.9
Gallbladder	100.0	100.0	0.0
Gynecologic	89.6	89.6	0.0
Hernial/peritoneal	100.0	100.0	0.0
Kidney, neoplastic	96.2	94.9	1.3
Liver/bile duct, neoplastic	97.0	98.5	–1.5
Lung/bronchus/larynx/oral cavity/nasopharynx	89.4	92.3	–2.9
Lymph node	97.1	97.8	–0.7
Prostate	93.4	92.9	0.5
Salivary gland	95.3	94.8	0.5
Skin	89.6	89.4	0.2
Soft tissue tumors	96.6	93.1	3.4
Stomach	92.4	93.6	–1.2
Overall	92.0	92.6	–0.6

**Figure 5:**
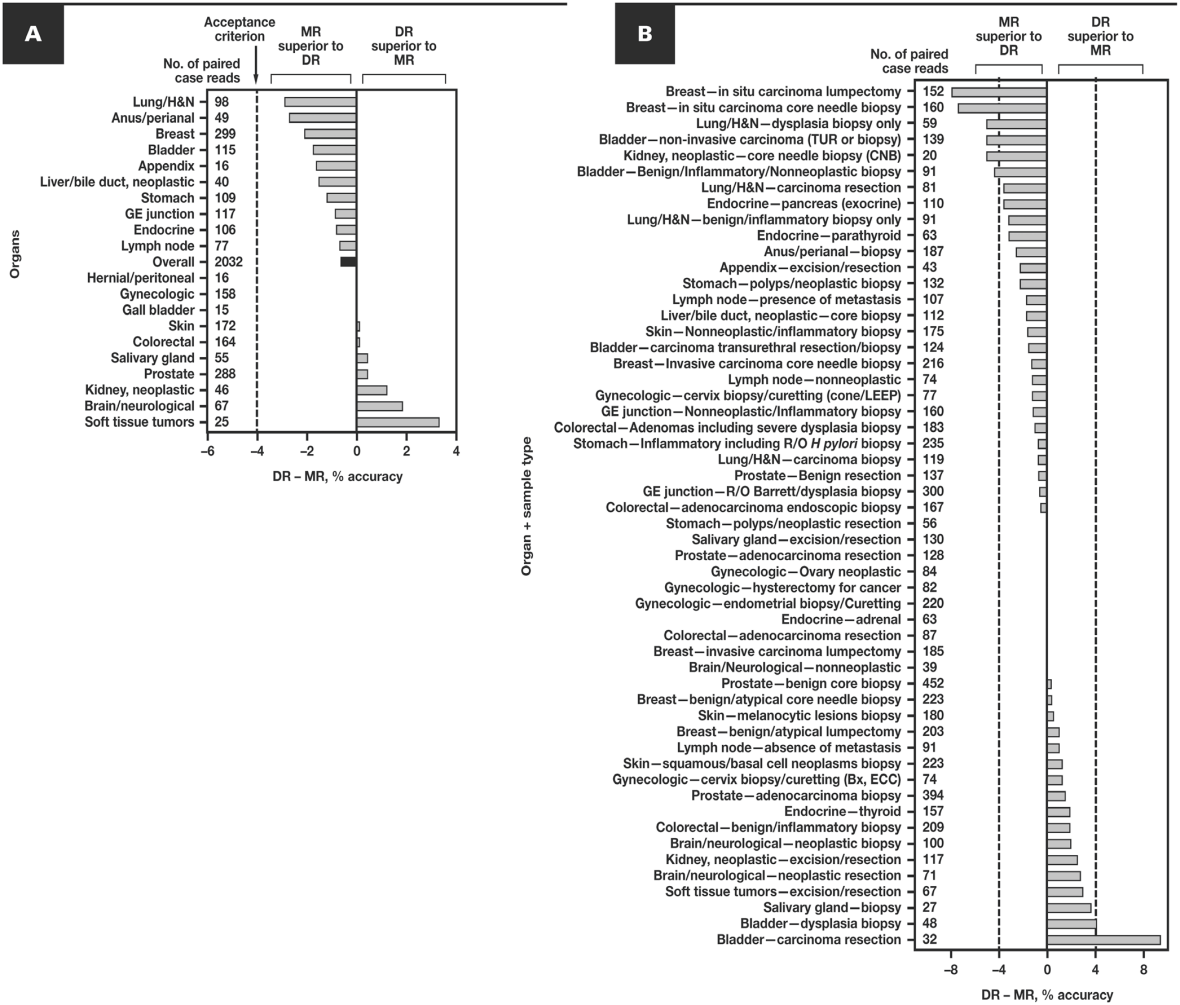
Percent differences in modality diagnostic accuracy by organ and sample type. **A**, Difference in diagnostic accuracy using DR vs MR across 20 organs, ranked from low to high, with the number of cases per organ group in the overall AEC cohort of 2032 cases. A DR – MR percent accuracy less than 0 indicates superiority of MR over DR, whereas a DR – MR more than 0 indicates superiority of DR over MR. The overall study acceptance criterion of –4% is indicated by a dotted line, with the number of study cases indicated by organ. **B**, DR – MR percent accuracy of 54 organ and sample types, ranked from low to high. Total number of paired modality (DR and MR) case reads is listed next to each organ and sample type (maximum of 4 per case, with 1 pair of reads for each of 4 pathologists per case). The remaining 20 organ + sample types and their DR – MR percent accuracy values are not plotted on the graph due to the low number of paired case reads and low number of major disagreements: Appendix—“not specified” and “other”: 20 case reads, 0 discrepancies; Gallbladder—all sample types: 60 case reads, 0 discrepancies; Gynecologic—“ovary benign/nonneoplastic”: 66 case reads, 0 discrepancies; Hernial/peritoneal—all subtypes: 62 case reads, 0 discrepancies; Kidney, neoplastic—“not specified” and “other”: 20 case reads, 0 discrepancies; Liver/bile duct, neoplastic—“wedge biopsy or resection”: 23 case reads, 0 discrepancies; Salivary gland—“CNB,” “FNA,” “not specified,” and “other”: 35 case reads, 0 discrepancies; Soft tissue tumors—“CNB,” “biopsy,” and “not specified”: 20 case reads, 2 discrepancies. AEC, all evaluable cases; Bx, biopsy; CNB, core needle biopsy; DR, digital read; ECC, endocervical curettage; FNA, fine-needle aspiration; GE, gastroesophageal; H&N, head and neck; LEEP, loop electrosurgical excision procedure; MR, manual microscopy read; R/O, rule out; TUR, transurethral resection.

All organ groups had DR – MR percent accuracy of –4% or more, although each organ group was underpowered to demonstrate statistical significance. DR – MR percent accuracy by organ group ranged from –2.9% (lung/head and neck) to +3.4% (soft tissue tumors) of case reads, with hernial/peritoneal, gynecologic, and gallbladder groups at 0% **Table 3**, **Figure 5A**. Among all 74 organ + sample type subgroups, DR – MR percent accuracy ranged from –7.89% (“breast—in situ carcinoma/lumpectomy”) to +9.38% (“bladder—carcinoma resection”) of paired (DR and MR) case reads in each subgroup, with 89% (66/74) of organ + sample type subgroups having DR – MR percent accuracy values within ±4% **Figure 5B**.

There were zero cases in which all 4 readers agreed by MR but disagreed by DR with the SD and vice versa. There were 2 cases in which 3 of 4 readers agreed by MR but disagreed by DR (case AX0005, cervix biopsy; case BZ0172, skin biopsy specimen with basal cell carcinoma) but 1 case in which 3 of 4 readers disagreed by MR but agreed by DR (case CZ0031, breast lumpectomy with intraductal papilloma). Remaining cases with 1 or 2 disagreements in DR but zero in MR, or vice versa, represented random occurrences, as seen in prior studies.^33,34^

### Root cause analysis of diagnostic discrepancies

We inspected all reader diagnoses to determine whether root causes of discrepancies were random occurrences, due to intrinsically challenging cases, or could be attributed to the digital pathology modality or system. We highlight the following organs with the lowest DR – MR percent accuracies by organ + sample type:

- *Breast*: Among all organ + sample type subgroups, 2 of 5 breast sample types (“in situ carcinoma—lumpectomy” and “in situ carcinoma—core needle biopsy”) had the lowest DR – MR percent accuracy **Figure 5B**. Paired case reads where the MR agreed, but the DR disagreed, with the SD (ie, “MR-agree/DR-disagree”) represent cases with potential DR-specific pitfalls. Among these 2 in situ carcinoma case types, 11 lumpectomy cases were represented in 11.8% (18/152) of case reads with MR-agree/DR-disagree, and 9 core needle biopsy cases were represented in 9.4% (15/160) of paired case reads with MR-agree/DR-disagree. For 8 of 15 of the core needle biopsy paired case reads, the DR diagnosis was less severe (ie, dysplasia or equivalent) than the SD, whereas none of the DR diagnoses were more severe (ie, invasive carcinoma or equivalent) than the SD, hinting at an association between DR and “undercalling” in situ carcinoma as dysplasia. The remaining 7 of 15 DR vs SD major discrepancy case reads were not related to severity along the dysplasia-carcinoma spectrum. However, this association was less consistent with lumpectomies, where 8 of 18 DR diagnoses were less severe, and 3 of 18 more severe, than the SD of in situ carcinoma. The other 3 breast sample types (encompassing benign and invasive carcinoma cases), by contrast, had very low DR – MR percent accuracy values **Figure 5B**, so the overall discrepancy rate for breast samples was driven in our study by slightly higher rates of DR disagreement for in situ carcinoma samples.
- *Lung*: The third lowest DR – MR percent accuracy was –5.08% for lung/head and neck, dysplasia biopsy specimens **Figure 5B**. The “MR-agree/DR-disagree” subset was 7 paired case reads from 6 different cases (out of 59 total paired case reads in that subgroup); here, 4 of 7 DR diagnoses were downgraded (ie, to reactive changes), but 3 of 7 were upgraded (to carcinoma in situ or more severe), suggesting no relationship between DR modality and over/undercalling.
- *Bladder*: The fourth and sixth lowest DR – MR percent accuracy, –5.04% and –4.40%, were the noninvasive carcinoma (transurethral resection or biopsy) and benign/inflammatory/nonneoplastic biopsy bladder sample types **Figure 5B**. Among the former subgroup, the “MR-agree/DR-disagree” subset was 9 cases, contributing to 11 of 139 paired case reads. Of these, 6 of 11 had a less severe diagnosis (such as “dysplasia”) with DR compared to the SD, but 4 of 11 had a more severe diagnosis (such as “invasive carcinoma”). Among the latter subgroup, the “MR-agree/DR-disagree” subset was 7 cases, contributing to 7 of 91 paired case reads. Of these, 6 of 7 had a more severe diagnosis (eg, “carcinoma in situ”) with DR compared to the SD, but none had a less severe diagnosis (such as “no abnormality”). Of the remaining 3 categories of bladder specimens, 2 categories (carcinoma resections and dysplasia biopsy specimens) had positive DR – MR percent accuracy values, suggesting that for those case types, the DR was *more* accurate than MR.
- *Kidney*: The fifth lowest DR – MR percent accuracy, –5.0%, was core needle biopsy **Figure 5B**, consisting of only 20 paired case reads. There were 2 paired “MR-agree/DR-disagree” case reads from 1 lymphoma case.
- *Stomach—inflammatory, including rule-out Helicobacter pylori biopsy*: Gastric biopsy cases (235 case reads) had an overall low DR – MR percent accuracy of –0.85%.

Our data show a low frequency of diagnoses discrepant with DR that are also known to be challenging with MR, such as dysplasia vs in situ carcinoma. We did not identify DR-modality specific root causes for these discrepancies, consistent with the overall very high percentage of scans considered by readers as acceptable for diagnosis.

### Comparison with other WSI system validation studies

**Figure 6:**
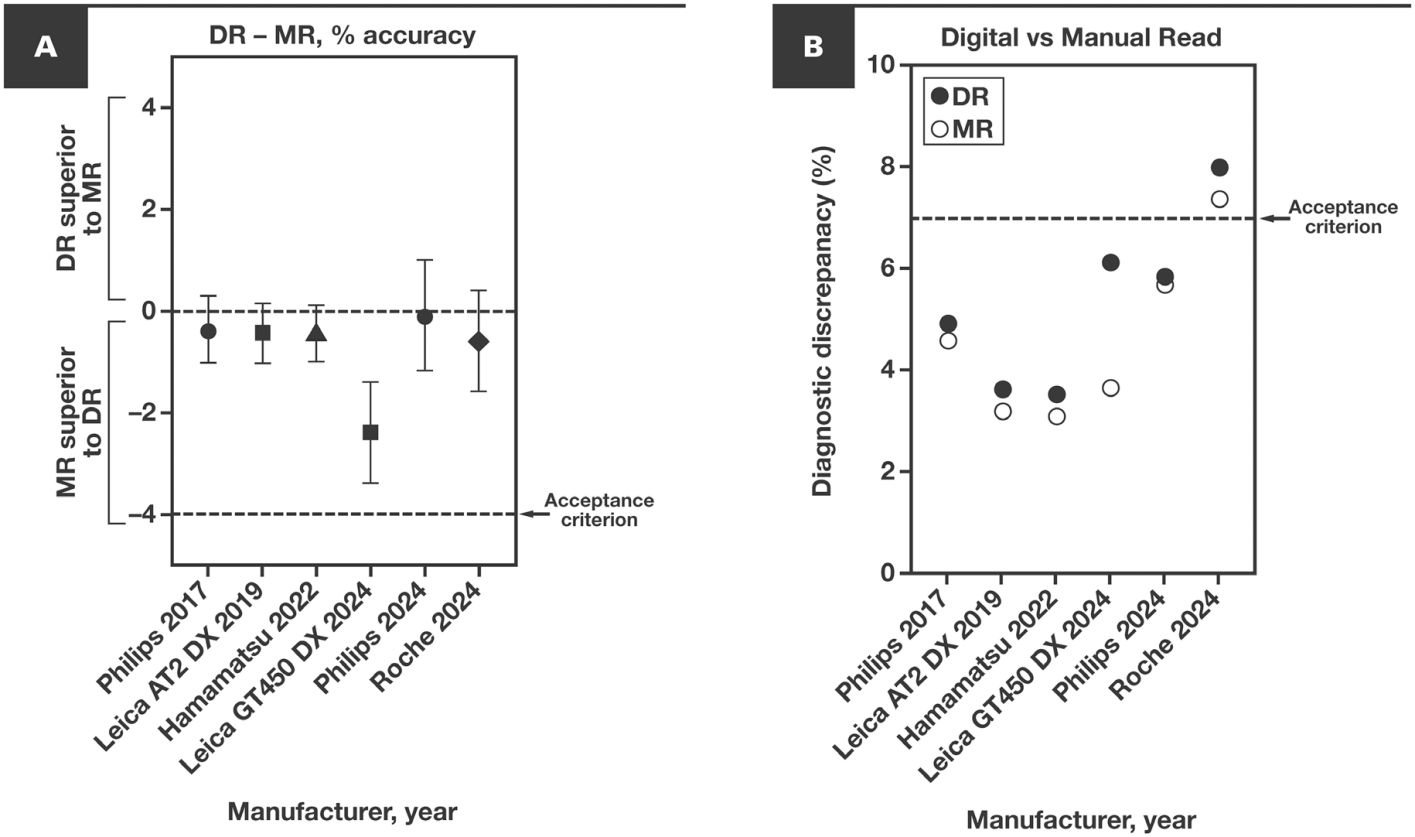
Comparison of MC study with prior WSI system studies. **A**, DR – MR percent accuracy for each FDA-cleared WSI system containing a unique scanner, by manufacturer and year of clearance, with bars representing ±95% CIs. Acceptance criterion for all studies was –4% or more for the lower bound of the 95% CI (dashed line). Note that all studies showed slight superiority of MR over DR, with overlap between the 95% CIs for the Roche study and all other studies. **B**, DR (solid circles) and MR (open circles) modality major diagnostic disagreements compared to SD for each WSI system/study. The DR acceptance criterion for other WSI system studies of 7% or less for the upper bound of 95% CI is indicated by the dashed line. Our study did not prespecify this acceptance criterion due to the noninferiority study design with matched manual microscopy comparator (MR). DR, digital read; FDA, US Food and Drug Administration; MC, method comparison; MR, manual microscopy read; SD, sign-out diagnosis; WSI, whole slide imaging.

Among ILR/precision studies in WSI system 510(k) clearances, the number of features ranged from 21 (Philips InteliSite Solutions)^28,32^ to 23 (Leica Biosystems Aperio AT2 DX and GT 450 DX and this study)^29,31,35-37^ to 30 (Hamamatsu Photonics NanoZoomer S360MD)^30^: a total of 44 different features in 1 or more studies and 8 features in all studies (although not all of these 8 features were tested at the same magnification) (Table S5). Each study had similar but distinct designs, and all achieved 85% or more precision in intersystem/site, intrasystem, and interreader metrics, with potential reasons for differences addressed in the Discussion. Among the MC/accuracy studies, the differences in DR vs MR modality accuracies were all similar (most with ~–1%), meeting the lower bound of CI acceptance criterion for noninferiority (≥–4.0%), although our modality-specific discrepancy rates were slightly higher than prior studies **Figure 6**.

#### Root cause of differences in MC/accuracy studies

**Figure 7:**
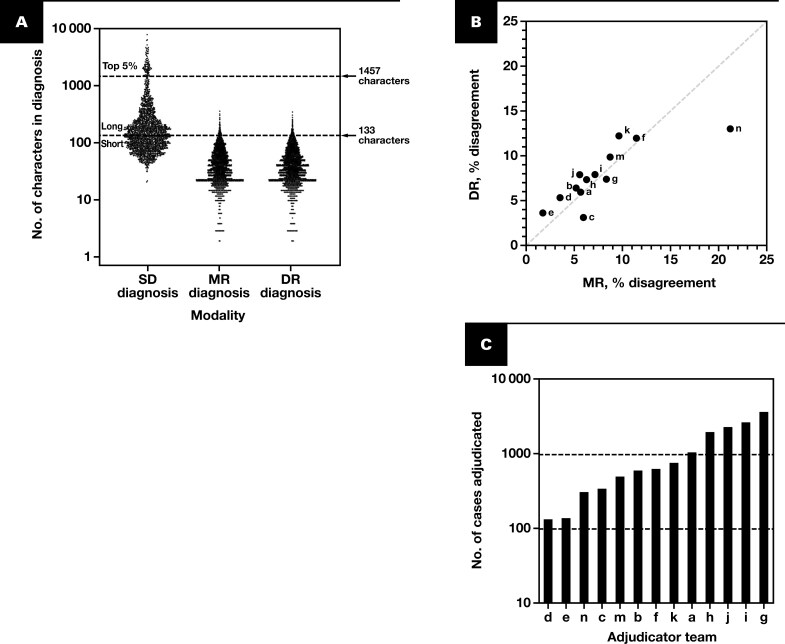
Length of diagnoses and adjudication team performance. **A**, Scatterplot of character lengths of 2032 input SD diagnoses (AEC population) and of readers’ 7562 MR and 7562 DR diagnoses plotted on log_10_ scale. Summary statistics are in **Table 4**. Note high similarity between MR and DR distributions (*P* = .684, paired *t* test of log-transformed data), compared to the much larger and wider distribution of SD (*P* < .001, paired *t* test with either MR or DR, log-transformed data). A logistic regression of SD distribution as a function of major vs nonmajor disagreement was statistically significant (*P* = .012), and the boundary between “long” (n = 936 cases) and “short” (1096 cases) SD diagnoses as 133 characters (dashed line) was defined in an unbiased fashion by maximization of Youden’s *J* statistic. Top 5% of longest SDs were more than 1457 characters (dashed line). **B**, Percent major diagnostic disagreements in each modality for 13 adjudication teams (teams arbitrarily designated by letters a-n, except “l”). **C**, Logarithmic plot of caseload by adjudicator team, ranked from low to high. AEC, all evaluable cases; DR, digital read; MR, manual microscopy read; SD, sign-out diagnosis.

Prior MC/accuracy studies specified that the upper bounds of the 95% CI of DR major disagreement compared to the original SD should be 7% or less. We questioned the relevance of this endpoint, taken in isolation, but also sought to understand the root cause of our results. We did not identify system-, site-, reader-, organ-, sample type-, or case diagnosis–related factors that accounted for higher discrepancy rates in both modalities (data not shown). However, the COVID-19 pandemic-associated resource constraints during the case screening/enrollment stage prevented all 4 study sites from condensing the primary SD from the medical record to allow efficient adjudication of SD vs DR or MR diagnoses, resulting in markedly different character length (including spaces) distributions for SD vs modality diagnoses **Figure 7A**, **Table 4**. Over half of cases (53.9%, 1096/2032 cases) had SD character lengths longer than the longest reader modality diagnosis in the entire study (354 characters for the single longest MR diagnosis). As expected, there was a weak positive correlation between SD character length and the time taken for readers to render a DR diagnosis (Spearman *r* = +0.2276, *P < *.0001, Figure S2), consistent with SD length being a proxy for case complexity. For further analysis, unbiased logistic regression identified “short” SDs as being 133 or fewer characters and “long” SDs as being 134 or more characters **Figure 7A**.

**Table 4:** Summary Statistics of Adjudicated Diagnoses

Diagnosis	Designation	Character lengths			
		n	Mean	Median	Range
Sign-out diagnosis	Top 5% of total	102	NA	NA	1455-7521
	Long (≥134)	936	511.9	235	134-7521
	Short (≤133)	1096	87.6	87	21-133
	Total	2032	87	142	21-7521
Digital read	Total	7562	44.6	34	2-343
Manual microscopic read	Total	7562	44.7	34	2-354

NA, not applicable.

Excessively long SDs were noted by several adjudicators as increasing burden when comparing diagnoses (data not shown). That our study engaged more adjudicators (20) than predicate device studies allowed us to test the hypothesis that excess SD length itself increased major disagreements. We compared major discrepancy rates across the 13 adjudicator teams, irrespective of the third adjudication (when needed). Adjudicator team major disagreement rates ranged from 3.7% to 13.1% for DR and 1.7% to 21.1% for MR, but consistent with aggregate data, for 10 of 13 teams, DR was greater than MR. Team “n” was an outlier in both modalities but represented only 2.1% (316/15 124) of all adjudications **Figure 7B**. Adjudicator teams managed a wide range of caseloads, from 131 cases (team “d”) to 3656 cases (team “g”) **Figure 7C**, but there was no relationship between modality discrepancies and team caseloads (data not shown).

**Figure 8:**
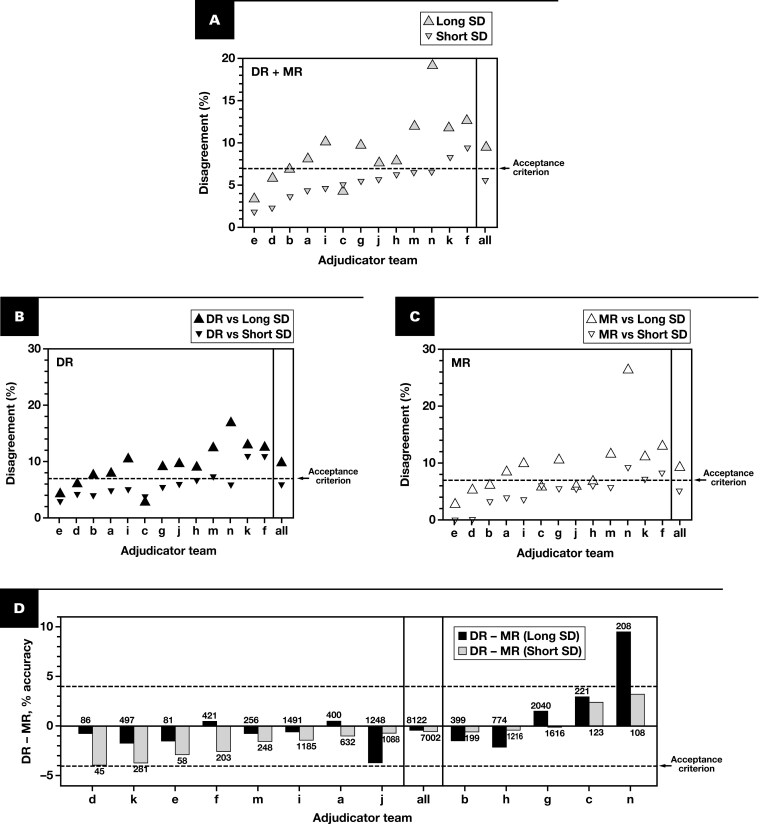
Diagnostic accuracy/discrepancy of “long” vs “short” reference SDs by adjudicator teams. **A**, Percent major disagreement rates of long (larger, up-pointing triangles) vs short (smaller, down-pointing triangles) SD for pooled DR and MR diagnoses by adjudicator team, ranked by disagreement of short diagnoses. Right-most lane is aggregate (“all”) data. **B**, **C**, Modality-specific major disagreement of long vs short SD by (**B**) DR and (**C**) MR modalities by adjudicator team, ranked as in **A**. In **A** to **C**, the horizontal dashed lines indicate an acceptance criterion for digital modality major disagreement rate in prior studies (7%). **D**, DR – MR percent accuracy of long and short SD by adjudicator team, ranked by value for short SDs. For reference, the overall study cohort lower bound of the 95% CI acceptance criterion of –4% or more is indicated by the dashed line. Number of paired (DR + MR) case reads on which each DR – MR percent accuracy is based, by adjudicator team and long vs short SD status, is indicated above or below each bar. DR, digital read; MR, manual microscopy read; SD, sign-out diagnosis.

Next, we used logistic regression to test the hypotheses that increased SD length was associated with case difficulty and/or adjudication difficulty. Across all (pooled MR + DR) modality reads, cases with short SDs had a major discrepancy rate of 5.48% vs 9.60% for long SD **Figure 8A**, with 12 of 13 adjudication teams having higher major discrepancy rates for long SD than for short SD in either modality **Figure 8B, C**. By contrast, all 13 adjudicator teams had DR – MR percent accuracy values of –4% or more for both long and short SDs **Figure 8D**. Our protocol specified that at least 10% of cases should constitute “difficult” cases, as defined in the predicate device study^34^ (Table S4); our study had a slightly higher representation, with 13.4% (2030/15 124 case reads) from such “difficult” cases **Table 5**. While the difference in proportion of difficult cases in long vs short SD groups was small (1.7%) **Table 5**, it was statistically significant due to large sample sizes (*P *= .002 by 2-sample test of proportions). Accordingly, we added an indicator variable, difficult vs nondifficult case, as a second covariate to our analysis. Difficult case status was not statistically significant between long SD and short SD populations (*P *= .25 by Wald test), and the regression coefficient and *P* value for SD length (represented as a continuous variable) both remained unchanged (data not shown). Thus, our data demonstrate a positive association between SD length and adjudication difficulty but not case difficulty per se.

**Table 5:** Proportion of “Difficult” Cases in Long vs Short Input SDs

Case reads	Short SD		Long SD		Total SD	
	n	%	n	%	n	%
“Difficult” cases	874	12.5	1156	14.2	2030	13.4
Non-“Difficult” cases	6128	87.5	6966	85.8	13 094	86.6
Total case reads	7002	100	8122	100.0	15 124	100.0

**Figure 9:**
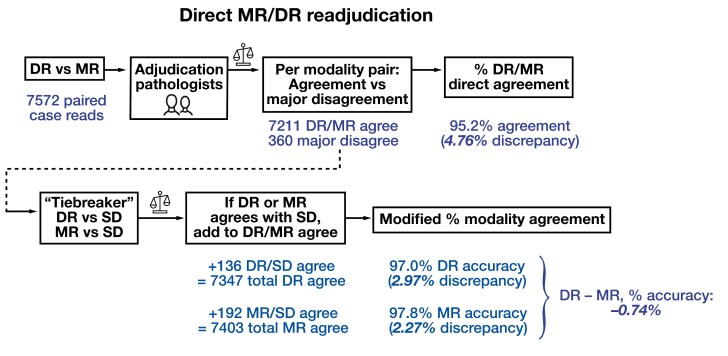
Direct (re)adjudication of reader diagnoses. Top row shows post hoc intermodality diagnosis adjudication workflow, with number of relevant paired case reads and summary metrics below. For the “tiebreaker” adjudication (second row), the 360 paired case reads with a major disagreement between DR and MR were separately adjudicated with the SD to calculate modified modality-specific discrepancy rates. DR, digital read; MR, manual microscopy read; SD, sign-out diagnosis.

Last, we directly (re)adjudicated DR with MR reader diagnoses in the AEC population of 2032 cases/7572 paired reads, initially blinded to SD, but then using the SD as a tiebreaker for major DR/MR disagreements. This post hoc analysis led to an intermodality disagreement rate of 4.77% **Figure 9**—markedly lower than when modality diagnoses were each compared to the (sometimes far longer) SD in the original adjudication. Using the SD as a tiebreaker, where possible, further reduced the DR disagreement rate to 2.97%, with a DR – MR percent accuracy of –0.74% **Figure 9**. These data confirm that discordance in the length of the diagnoses to be compared increased the burden of case adjudication, leading to slightly higher major disagreement rates compared to prior studies, irrespective of reading modality.

## DISCUSSION

Many studies have demonstrated that digital pathology is noninferior to manual microscopy for diagnosis (as mentioned in the Introduction and previously reviewed by Azam et al^40^). As of November 2024, just 6 WSI systems with unique scanners had received FDA 510(k) clearance: Philips InteliSite Pathology Solution in 2017^28^ and v5.1 in 2024,^32^ Leica Biosystems Aperio AT2 DX in 2019^29^ and GT 450 DX in 2024,^31^ Hamamatsu Photonics NanoZoomer S360MD in 2022,^30^ and, based on the work reported here, RDPD^37^ on June 14, 2024, and December 16, 2024.^37,38^ All 6 of these systems were tested using similarly designed performance studies,^24^ and all have yielded highly similar results, suggesting that any differences are due to study cohort and/or execution rather than the quality of WSI systems tested.

Both the Leica AT2 DX and Roche RDPD ILR/precision studies identified “necrosis,” “osteoclasts,” and “nuclear grooves” as among the most challenging features to identify.^35^ In these 2 studies, slides were scanned to generate WSIs at the same magnification as the feature was intended to be read (ie, at 20× or 40×). In a follow-on Leica precision study using GT 450 DX, all WSIs were scanned at 40×, but the 20× features were projected on the display monitor at half of the 40× source WSI resolution.^36^ The GT 450 DX study also identified “necrosis” and “osteoclasts” as challenging features to identify but also identified “hemosiderin,” “adipocytes,” and “pleomorphic nucleus of malignant cell” as challenging,^36^ whereas in our RDPD study, the latter 2 features were not challenging, being identified in 99.5% and 100% of 144 study reads for each feature, respectively **Figure 2**. While the 20× feature “osteoclasts”—multinucleated cells that resorb osteoid matrix—can resemble another feature on the list, “foreign body giant cells,” these 2 features are normally found in vastly different contexts. Accordingly, misidentified “osteoclast” ROIs in the RDPD study were most commonly called “osteocytes” because they are both found in bone, but the latter was not listed by the screening pathologist as the ROI’s primary feature. We noted a similar trend with “foreign body giant cells” vs “granulomas.” The Leica AT2 DX study highlighted that recognition of “nuclear grooves” depends on association with a diagnostic entity such as papillary carcinoma of the thyroid, where they are a common “textbook” feature.^35^ A “nuclear grooves” case in our study with a very low identification rate was LCH, which was most commonly (mis)identified as a “Reed-Sternberg cell,” suggesting readers misidentified the context as Hodgkin disease, rather than failing to recognize the feature per se. Notably, the Leica GT 450 DX study did not identify “nuclear grooves” as a challenging feature, despite the fact that 1 of the “nuclear grooves”–containing cases was a “Langerhans cell granuloma.”^36^ Both the Leica AT2 DX and GT 450 DX studies illustrated the challenge in identifying a noncanonical Reed-Sternberg cell by H&E that, in a real-world setting of Hodgkin disease, would be subject to confirmatory IHC.^35,36^ In contrast, our RDPD study had mostly canonical-appearing Reed-Sternberg cells with only a 6.5% (14/216 reads) discrepancy rate.

Our data indicate that a feature and its histopathologic context are inseparable. The specific case and feature example included in the study cohort can have a substantial impact on the overall precision of that feature, and differences in the rate of identification of just a few features can affect overall study precision. In addition, the study design does not actually measure “precision” because it penalizes reproducible yet incorrect feature identification. This is due to screener selection bias of a particular case/ROI that includes the feature and a particular feature in a ROI when additional listed features might be present. Error increases if the reader does not take the time to identify all listed features in the ROI. The fact that our readers sometimes failed to identify the primary feature in ROIs when multiple listed features were present supports the hypothesis that reader impatience might contribute to errors, especially in highly powered studies that require repetitive feature-hunting tasks. Finally, despite each ILR/precision study consisting of thousands of ROI read comparisons, the feature lists tested represent only a tiny fraction of the “feature space” pathologists need to master for primary diagnosis.

Comparison of the 6 large WSI system MC/accuracy studies used for 510(k) clearance of WSI systems with unique scanners revealed a small yet remarkably consistent difference in the accuracy of digital vs manual microscopy diagnoses (typically ~1%) compared to the noninferiority margin (4%) **Figure 6**. Interestingly, manual microscopy showed slightly higher point estimates of overall accuracy (ie, a lower major disagreement rate) than digital reads *in all 6 studies*. We extended this observation to the adjudication process among the large pool of adjudicators in our study. In addition to the fact that all SDs were originally rendered using a microscope, all study pathologists use light microscopy in their daily sign-out, making them likely to be more accurate with that modality. However, many organ/sample types in our study were read slightly more accurately digitally than with microscopy **Figure 5**. The very low percentage of slides where pathologists requested a higher magnification scan (40×) to render a diagnosis (6/3259 slides; 0.18%) supports the hypothesis, tested in the Leica AT2 DX predicate device study, that 20× WSIs are adequate for most primary diagnoses.^34^ In contrast, in the Leica GT 450 DX study, all slides were scanned at 40×, yet there was still a similar (small) difference in modality diagnostic accuracy,^36^ suggesting little to no incremental gain with upfront higher magnification scanning for primary diagnosis.

MC studies are designed to identify method-specific pitfalls. While manual microscopy is considered the gold standard, some organ, sample, or case types are intrinsically challenging and prone to diagnostic disagreement and would thus be expected to have similar disagreement rates in both modalities. Nevertheless, some sample types and diagnoses can be potentially more challenging in digital vs microscope formats, such as where very crisp images are required to accurately characterize fine features like chromatin patterns (eg, dysplasia vs carcinoma in situ) or infectious agents (eg, *H pylori* in stomach biopsy specimens), or where the pathologist might adjust focus at high magnification to better visualize objects at variable tissue depth (eg, special stains for infectious agents, such as a fast-red stain for *Mycobacteria*).^40-42^

Our MC study using RDPD showed the expected low major diagnostic disagreement rates across organs and sample types, with some having slightly higher accuracy with MR and others higher accuracy with DR. Notably, among all 74 organ and sample type subgroups in our study, 2 of 5 breast categories (lumpectomies and core needle biopsy specimens with in situ carcinoma) had the highest proportion of discrepancies in DR compared to MR, with a slight preponderance of lesions undercalled using DR, consistent with known diagnostic challenges associated with accurate diagnoses within the dysplasia-carcinoma spectrum.^43^ However, the other 3 of 5 breast sample subgroups did not show this trend, and we did not observe a consistent DR-specific trend to upgrade or downgrade diagnoses in any of the other organs or sample types. A prior study using digital pathology identified a slight preference for overcalling breast lesions,^44^ but later studies have documented an association of DR with slight undercalling of breast lesions,^45,46^ with the latter study attributing differences to reporting rather than the digital modality.

Slightly higher major discrepancy rates for both modalities in our RDPD MC study raised the question of the root cause of differences compared to prior studies. We pinpointed the study contracting phase, which commenced during the early stages of the COVID-19 pandemic: all 4 study sites did not condense input SDs to lengths comparable to those that study readers might generate. Our analysis supports the hypothesis that excessively long SDs increased adjudication burden, resulting in higher major disagreement rates for cases with long SD diagnoses, independent of modality and case difficulty (as defined in the Leica AT2 DX predicate device study^34^), across most adjudication teams. This hypothesis was supported by direct readjudication of MR vs DR diagnoses, which, when using the SD as a tiebreaker, reduced the DR-specific diagnostic discrepancy rate below 3% **Figure 9**. Our results are consistent with a recent study showing higher agreement rates with direct DR/MR adjudication than when either was compared to SD.^46^

Our results add to the growing body of evidence that 20×-magnification WSIs are largely equivalent to manual microscopy for most tissues and diagnostic scenarios while offering advantages to laboratories by reducing scan times, storage requirements, and image retrieval/screen refresh times; higher-magnification 40× WSIs may only be required for a subset of challenging cases or case types.

Finally, taken together with prior studies, our results question the ongoing regulatory requirement for large-scale clinical performance studies of WSI systems for primary diagnosis. Importantly, pathologists are required to judge tissue and staining quality irrespective of modality, as well as WSI quality, and validate specific use cases in qualified laboratories as part of professional practice and laboratory accreditation.^47^ Similar results from resource-intensive ILR/precision and MC/accuracy studies of 6 distinct WSI systems supporting 510(k) clearance for primary diagnosis, coupled with increased confidence in WSI-based diagnoses in general, raise the question of whether less burdensome study designs that emphasize usability and analytical evidence of image quality would be sufficient to ensure WSI system safety and effectiveness.

In conclusion, RDPD demonstrates clinical performance comparable to manual microscopy for primary diagnosis in surgical pathology.

## Supplementary material

Supplementary material is available at *American Journal of Clinical Pathology* online.

aqaf052_suppl_Supplementary_Tables_S1-S5_Figures_S1-S2

## Data Availability

Source data and analysis are on file with Roche Diagnostics and with the US Food and Drug Administration. Raw study diagnoses are confidential due to their status as Protected Health Information under the Health Insurance Portability and Accountability Act of 1996. Requests concerning data or analyses can be directed to rotkreuz.datasharingrequests@roche.com for consideration.

## Note

While this manuscript was in final review, on February 28, 2025, a seventh WSI system with a unique scanner (Epredia E1000) received FDA 510(k) clearance (K241717). In their accuracy study, the point estimate case major discrepancy rate was 2.54% in the digital modality and 2.65% in the microscopic modality, a difference of 0.11%, which is consistent with the data in **Figure 5** showing that, when considering the 95% CIs, the differences between all but one of the systems and manual microscopy overlap with zero.
