# Correction to: Roche Digital Pathology Dx whole slide imaging system is comparable to traditional microscopy for primary diagnosis in surgical pathology

**DOI:** 10.1093/ajcp/aqaf076

**Published:** 2025-06-28

This is a **correction** to:

Keith A Wharton, Jim Ranger-Moore, Hon Seng, Alexander D Borowsky, Cynthia A Behling, Nicolas Cacciabeve, Michael LaFriniere, Richard M Feddersen, Crystal Williams, Drew Baldwin, Richard Louie, Lauren Murata, Cameron Smith, Andrea Visoski, Mingfei Zhao, Shalini Singh, Tracie N Gardner, Roche Digital Pathology Dx whole slide imaging system is comparable to traditional microscopy for primary diagnosis in surgical pathology, *American Journal of Clinical Pathology*, 2025;, aqaf052, https://doi.org/10.1093/ajcp/aqaf052

In the originally published version of this manuscript, author Richard M Feddersen’s credentials were incorrectly listed as “MD, PhD”, instead of “MD”.

This error has been corrected.

22 September 2025 This correction notice has been revised to address an earlier error concerning author Keith A Wharton’s credentials. The author’s credentials were previously modified in error and have now been corrected in the article to read “MD, PhD”.

