# Supplementary material for: Roche Digital Pathology Dx whole slide imaging system is comparable to traditional microscopy for primary diagnosis in surgical pathology
Source: Am J Clin Pathol. 2025 Jun 10;164(3):367–84. doi: 10.1093/ajcp/aqaf052 (PMC12421238; doi:10.1093/ajcp/aqaf052)
Supplement: aqaf052_suppl_Supplementary_Tables_S1-S5_Figures_S1-S2 [file aqaf052_suppl_supplementary_tables_s1-s5_figures_s1-s2.pdf]

Supplementary Figure S1 Case disposition and population definitions for method comparison study.

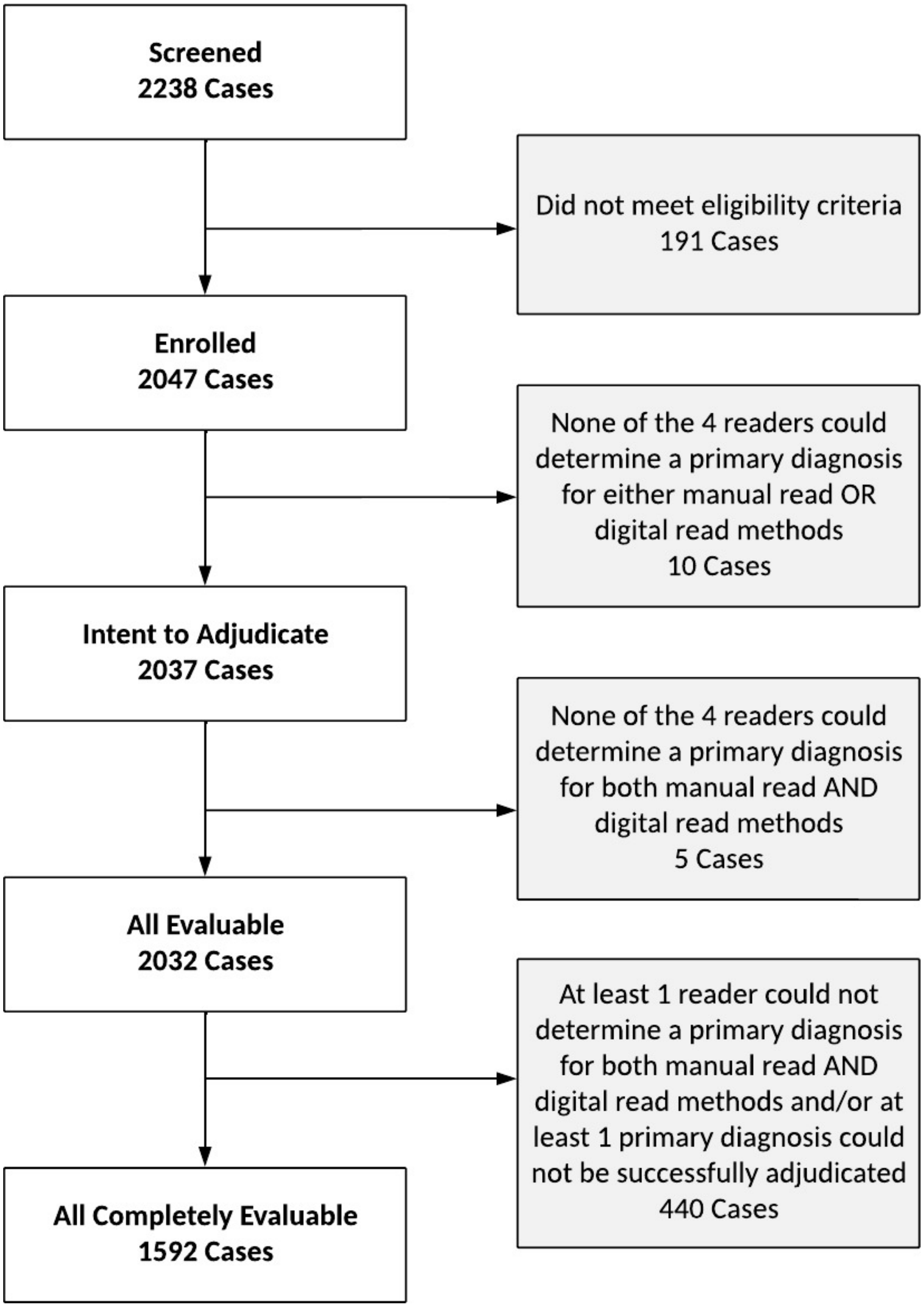

Supplementary Figure S2 Correlation between SD character length and time to DR diagnosis.

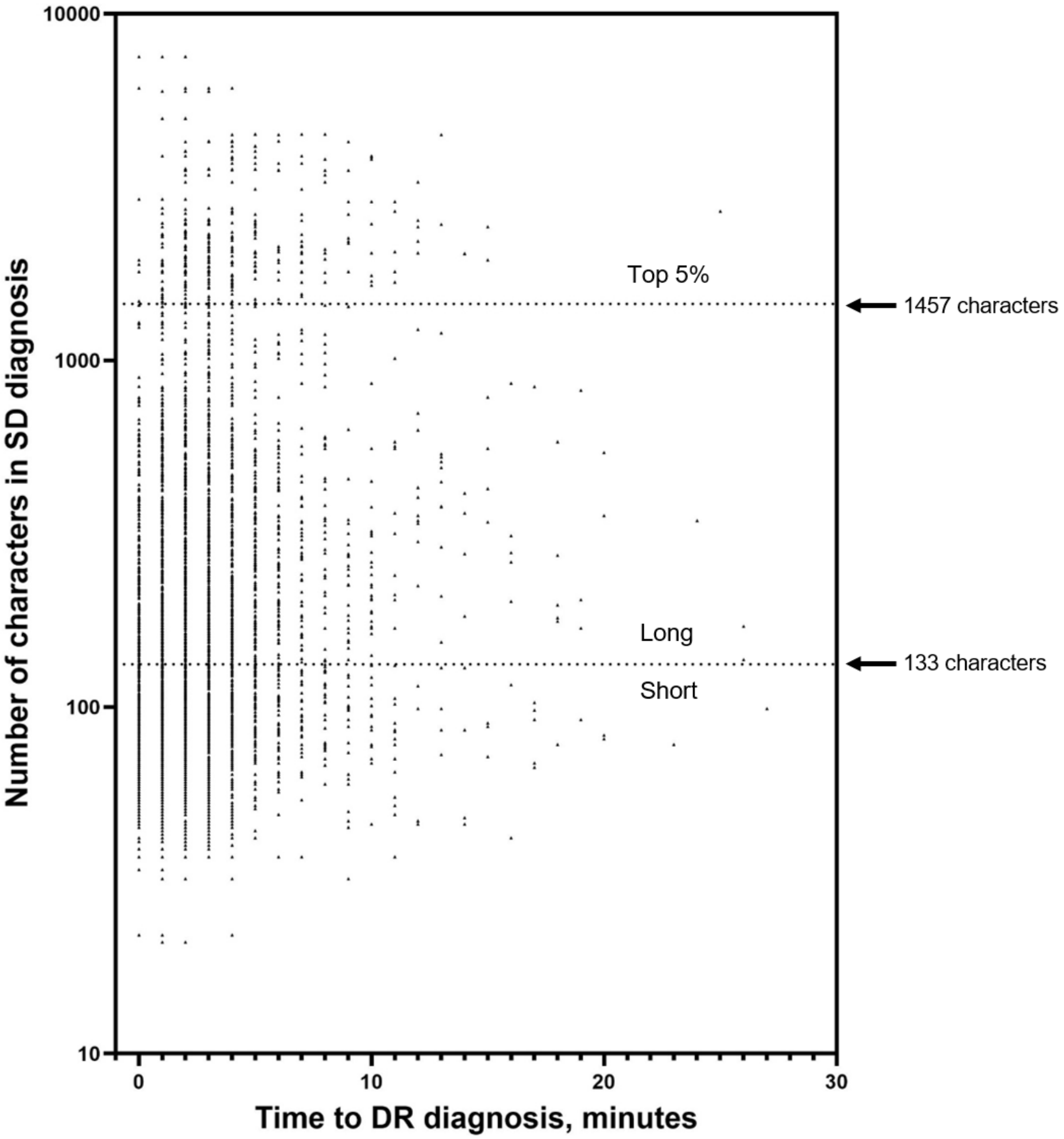

Log_{10} plot of number of characters in reference SD diagnosis vs time for each reader to render a DR modality diagnosis. Excludes 26/7562 (0.34%) case reads in the "all evaluable cases" (AEC) subpopulation that took >30 minutes to render a diagnosis, presumably due to leaving the system while logged onto a case. Horizontal dashed lines indicate long vs short diagnosis and top 5% of SD lengths from **FIGURE 6A**. Spearman *r* correlation = 0.2276, with 2 tailed $P < .0001$). Time to DR diagnosis was calculated from timestamp differences between starting and completing the case, measured to nearest minute (cases that took 0 minutes to read were diagnosed in less than 1 minute). DR, digital read; SD, signout diagnosis.

Supplementary Table S1 Sites and principal investigators for each study

	Study	
**Site**	**ILR/Precision**	**MC/Accuracy**
PI: Alexander D. Borowsky, MD, PhD<br>University of California Davis Health<br>440 V Street<br>Sacramento, CA 95817	Site A	Site D
PI: Cynthia Behling, MD, PhD<br>Pacific Rim Pathology<br>9295 Farnham St., Suite 100<br>San Diego, CA 92123	Site B	Site B
PI: Nicolas Cacciabeve, MD<br>Advanced Pathology Associates<br>9901 Medical Center Dr.<br>Rockville, MD 20850	Site C	Site C
PI: Richard M. Feddersen, MD, PhD<br>Tricore Reference Laboratories<br>1001 Woodward Place NE<br>Albuquerque, NM 87102	n/a	Site A

Abbreviations: ILR, interlaboratory reproducibility; MC, method comparison; PI, principal investigator.

Supplementary Table S2 Precision/ILR study feature list and tissue sample sources

Primary Feature (Magnification Level [a])	Organ Systems/Tissue Types of the 3 Enrolled Study Cases [b]		
Chondrocytes (20x)	Bone (left proximal humerus)	Bone (right scapula)	Soft tissue (chest, xiphoid)
Fat cells (adipocytes) (20x)	Lymph node (anterior prostatic)	Lymph node (pelvis, right)	Omentum
Foreign body giant cells (20x)	Breast, lower outer	Liver #1	Soft tissue (sixth intercostal muscle
Goblet cells (20x)	Colon (ascending)	Duodenum (second portion)	Lung (left upper lobe)
Granulomas (20x)	Lung (left lower lobe)	Lymph node (inguinal)	Lymph node (right axillary)
Infiltrating or metastatic lobular carcinoma (20x)	Breast (left)	Breast (right)	Chest wall (right)
Intraglandular necrosis (20x)	Breast (left)	Breast (right, lower outer quadrant)	Oral cavity (left buccal mucosa)
Osteoclasts (20x)	Bone (fibula, proximal, lesion, right)	Bone (left temporal bone)	Bone (tibia, left)
Osteocytes (20x)	Bone (frontal, right)	Bone (right tibia)	Pelvis (left, acetabular lesion)
Pleomorphic nucleus of malignant cell (20x)	Brain (right temporal mass)	Soft tissue (left thigh)	Soft tissue (right thigh)
Serrated intestinal epithelium (eg, sessile serrated polyp) (20x)	Colon (ascending, polyp)	Colon (ascending, polyp)	Colon (ascending, polyp)
Skeletal muscle fibers (20x)	Breast (left)	Left thigh	Thyroid gland
Asteroid bodies (40x)	Knee, right, synovium #3	Lung (upper lobe)	Lymph node
Clear cells (40x)	Aorta, inter aorta caval lymph node	Left kidney	Ovary and fallopian tube
Foreign bodies (eg, plant material or foreign debris) (40x)	Aorta, ascending, pseudoaneurysm wall	Small intestine and colon	Soft tissue (abdomen)
Hemosiderin (pigment) (40x)	Breast (left chest wall nodule)	Breast (right)	Breast (right)
Megakaryocytes (40x)	Bone (distal sternum and right ribs)	Bone (rib, right)	Buttock (left, lesion)
Necrosis (40x)	Lung (right lower lobe)	Lung (right upper lobe)	Right great toe
Nerve cell bodies (eg, ganglion cells) (40x)	Colon (cecum, polyp x2)	Esophagus	Soft tissue (left paraspinal)
Nuclear grooves (40x)	Bone (left acetabulum)	Left fallopian tube and left ovary	Thyroid (right)
Osteoid matrix (40x)	Bone (left acetabulum)	Bone (right tibia)	Bone (right ulna)
Psammoma bodies (40x)	Brain (posterior fossa tumor)	Brain (right frontal tumor)	Thyroid (lobe, left)
Reed-Sternberg cell (40x)	Lymph node (cervical right, level IV)	Neck mass (right)	Thymus

Source: Listing 16.2.1 (Appendix 16.2)
[a] Protocol-specified magnification level to be used for VENTANA DP 200 scanning of the given primary feature.
[b] As designated by the Screening Pathologist on the screening CRF.

Abbreviation: ILR, inter-laboratory reproducibility.

Supplementary Table 3 Accuracy/MC study organ and target number of case subtypes for enrollment

ORGAN	# Total Cases (*N*) [a]	#Subtype Cases (*n*)	SUBTYPES (procedures)
**Breast**	**300**	50	Benign/ Atypical Core Needle Biopsy
		50	Benign/ Atypical Lumpectomy
		50	In-Situ Carcinoma Core Needle Biopsy
		50	In-Situ Carcinoma Lumpectomy
		50	Invasive Carcinoma Core Needle Biopsy
		50	Invasive Carcinoma Lumpectomy
**Prostate**	**300**	120	Benign Core Biopsy
		30	Benign Resection
		120	Adenocarcinoma Biopsy
		30	Adenocarcinoma Resection
**Lung/ Bronchus/ Larynx/ Oral Cavity/ Nasopharynx**	**100**	25	Benign/ Inflammatory Biopsy Only
		25	Dysplasia Biopsy Only
		30	Carcinoma Biopsy
		20	Carcinoma Resection
**Colorectal**	**150**	50	Benign/ Inflammatory Biopsy
		50	Adenomas Including Severe Dysp Biopsy
		40	Adenocarcinoma Endoscopic Biopsy
		10	Adenocarcinoma Resection
**GE Junction**	**100**	50	R/O Barrett/ Dysplasia Biopsy
		50	Non-Neoplastic/Inflammatory Biopsy
**Stomach**	**100**	50	Inflammatory Including R/O *H. pylori* Biopsy
		40	Polyps/ Neoplastic Biopsy
		10	Polyps/ Neoplastic Resection
**Skin**	**175**	50	Non-Neoplastic/ Inflammatory Biopsy
		50	Squamous/ Basal Cell Neoplasms Biopsy
		75	Melanocytic Lesions Biopsy
**Lymph Node**	**100**	75 (1/2 ea +/ neg)	For Presence/ Absence of Metastasis (*No micrometastases smaller than 0.5 mm*)
		25	Non-Neoplastic
**Bladder**	**100**	25	Benign/ Inflammatory/ Non-Neoplastic Biopsy
		25	Dysplasia Biopsy
		25	Noninvasive Carcinoma (Transurethral Resection or Biopsy)
		15	Carcinoma Transurethral Resection/Biopsy
		10	Carcinoma Resection
**Gynecological**	**150**	50	Endometrial Biopsy/ Curetting
		20	Cervix Biopsy/ Curetting (Bx, ECC)
		20	Cervix Biopsy/ Curetting (Cone/LEEP)
		20	Ovary Benign/Non-Neoplastic
		20	Hysterectomy for endometrial or cervical cancer
		20	Ovary Neoplastic
**Liver/Bile Duct, Neoplastic**	**50**	40	Core Biopsy
		10	Wedge Biopsy or Resection
**Endocrine**	**100**	50	Pancreas (exocrine)
		30	Thyroid
		10	Parathyroid
		10	Adrenal
**Brain/ Neurological**	**60**	10	Non-Neoplastic
		25	Neoplastic Biopsy
		25	Neoplastic Resection
**Kidney, Neoplastic**	**50**	50	All Comers (Consecutive Cases)
**Salivary Gland**	**50**	50	All Comers (Consecutive Cases)
**Hernial/ Peritoneal**	**15**	15	All Comers (Consecutive Cases)
**Gallbladder**	**15**	15	All Comers (Consecutive Cases)
**Appendix**	**15**	15	All Comers (Consecutive Cases)
**Soft Tissue Tumors**	**20**	20	All Comers (Consecutive Cases)
**Anus/ Perianal**	**50**	50	Biopsy

ECC = endocervical curettage; LEEP = loop electrosurgical excision procedure; R/O = rule out.
[a] The number of cases will be divided across four sites. The distribution of the types of cases between the sites will be similar.

Supplementary Table 4 Difficult vs non-difficult case diagnosis designations by organ
Case diagnoses designated as "difficult" are indicated in bold with an asterisk. Target case enrollment is indicated next to each organ type.

Breast: ~300 Cases

I. Reactive/Inflammatory
    a. **Biopsy site changes, negative for malignancy**∗
    b. Fat necrosis
    c. Duct ectasia
    d. Gynecomastia
II. Non-proliferative ductal lesions
    a. Cyst
    b. Apocrine metaplasia
III. Adenosis/sclerosis lesions
    a. **Sclerosing adenosis**∗
    b. **Radial scar/complex**∗
IV. Fibroepithelial lesions
    a. Fibroadenoma
    b. Tubular adenoma
    c. **Lactating adenoma**∗
    d. Mammary hamartoma
    e. **Phyllodes tumor—benign/borderline/low**∗
    f. **Phyllodes tumor—malignant**∗
V. Lobular in situ lesions
    a. LCIS (lobular carcinoma in situ)
    b. Atypical lobular hyperplasia
    c. Columnar cell lesions and flat epithelial atypia
    d. Columnar cell change (no atypia)
    e. Flat epithelial atypia
VI. Intraductal proliferative lesions
    a. Usual ductal hyperplasia
    b. Atypical ductal hyperplasia
    c. Intermediate grade DCIS negative for invasion
    d. **High grade DCIS negative for invasion**∗
VII. Papillary lesions
    a. Intraductal papilloma (no atypia or DCIS)
    b. **Sclerosing papilloma**∗
    c. Encapsulated papillary carcinoma
    d. **Ductal adenoma**∗
    e. **Collagenous spherulosis**∗
VIII. Nipple
    a. Nipple adenoma
    b. **Paget's disease**∗
    c. Squamous metaplasia of lactiferous ducts
IX. Invasive carcinoma, common types
    a. Invasive ductal carcinoma
    b. Invasive mammary carcinoma NOS
    c. **Invasive lobular carcinoma***
    d. Tubular carcinoma
    e. **Invasive cribriform carcinoma***
    f. **Mucinous carcinoma***
    g. Invasive micropapillary carcinoma
X. Other applicable diagnoses (*Note: Reasonable efforts should be made to align with above diagnostic options for this organ category prior to the selection of this option.)*

Prostate: ~300 Cases

I. Benign nodular hyperplasia
II. PIN
III. **Prostatic adenocarcinoma, low grade (Total Gleason score 6 or less)***
IV. Prostatic adenocarcinoma, high grade (Gleason score 7 or greater)
V. **Nephrogenic adenoma***
VI. Xanthoma
VII. Other applicable diagnoses (*Note: Reasonable efforts should be made to align with above diagnostic options for this organ category prior to the selection of this option.)*

Lung/ Bronchus/ Larynx/ Oral Cavity/ Nasopharynx: ~100 Cases

I. Inflammatory
   a. Usual interstitial pneumonia
   b. **Noncaseating (non-necrotizing) granulomas c/w sarcoid***
   c. Granulomas with evidence of infection
   d. Langerhans cell histiocytosis
II. Neoplastic
   a. Squamous cell carcinoma in situ
   b. Invasive squamous carcinoma
   c. Adenocarcinoma
   d. Adenocarcinoma in situ
   e. Small cell carcinoma
   f. **Large cell neuroendocrine carcinoma***
   g. Carcinoid tumor
   h. Pulmonary hamartoma
   i. **Solitary fibrous tumor***
   j. **Lymphoma***
   k. Minute pulmonary chemodectoma
   l. Granular cell tumor
   m. **Nodular pulmonary amyloidosis***
III. Other applicable diagnoses (*Note: Reasonable efforts should be made to align with above diagnostic options for this organ category prior to the selection of this option.)*

Colorectal: ~150 Cases

I. **Active idiopathic inflammatory bowel disease***
II. Focal active colitis
III. Tubular adenoma
IV. Adenoma with high grade dysplasia
V. Invasive adenocarcinoma
VI. Hyperplastic polyp
VII. **Granulomatous gastritis***
VIII. Lymphocytic esophagitis or gastritis
IX. **Graft-versus-host disease***
X. **Unusual polyp variants (eg, Peutz-Jeghers)***
XI. **Infections (eg, *Helicobacter*, *Giardia*, fungal)***
XII. **Distinctions between various types of IBD (eg, Crohn disease, ulcerative colitis, ischemic or infectious colitis)***
XIII. Neuroendocrine tumor
XIV. Well-differentiated mucinous tumors
XV. Microscopic colitis
XVI. Collagenous colitis
XVII. Other applicable diagnoses (*Note: Reasonable efforts should be made to align with above diagnostic options for this organ category prior to the selection of this option.)*

GE Junction/Esophagus: ~100 Cases

I. Esophagitis
II. Eosinophilic esophagitis
III. Barrett esophagus, negative for dysplasia
IV. **Barrett esophagus, indefinite for dysplasia***
V. **Barrett esophagus, with low grade dysplasia***
VI. **Barrett esophagus, with high grade dysplasia***
VII. **Low grade squamous dysplasia***
VIII. High grade squamous dysplasia
IX. **Intramucosal (or in situ) adenocarcinoma (biopsy)***
X. **Intramucosal (or in situ) adenocarcinoma (resection)***
XI. Invasive squamous carcinoma
XII. Invasive adenocarcinoma
XIII. Other applicable diagnoses (*Note: Reasonable efforts should be made to align with above diagnostic options for this organ category prior to the selection of this option.)*

Stomach: ~100 Cases

I. Chronic and/or active gastritis
II. Chronic gastritis
III. Chronic gastritis with intestinal metaplasia
IV. **Intestinal metaplasia with low grade dysplasia***
V. High grade dysplasia
VI. **Adenocarcinoma, NOS (in biopsy)***
VII. Invasive adenocarcinoma
VIII. **Hyperplastic gastric polyp***
IX. **Leiomyoma***
X. **Duodenum- Celiac disease***
XI. **Duodenum- Duodenitis***
XII. **Duodenum- Duodenal adenoma***
XIII. Other applicable diagnoses (*Note: Reasonable efforts should be made to align with above diagnostic options for this organ category prior to the selection of this option.)*

Skin: ~175 Cases

I. Inflammatory
    a. Active inflammation
    b. Folliculitis
    c. Pyogenic granuloma
    d. **Impetigo***
    e. Molluscum contagiosum
    f. Chronic inflammation
    g. **Bite or Sting***
    h. Lichen planus
    i. **Graft v Host Disease***
    j. Psoriasis
    k. **Pemphigus vulgaris***
II. Fungal infections
    a. **Candidiasis***
III. Benign neoplasms
    a. Acrochordon
    b. Actinic keratosis
    c. Hemangioma
    d. Common wart
    e. Condyloma acuminatum

f. Cyst, epidermoid
g. Dermatofibroma
h. Keloid
i. Keratoacanthoma
j. Lentigo
k. Mole
l. Molluscum contagiosum
m. Sebaceous hyperplasia
n. Seborrheic keratosis

IV. Malignant neoplasms
a. Basal cell carcinoma
b. Melanoma
c. Melanoma in situ
d. Sebaceous carcinoma
e. Squamous cell carcinoma
f. Basal cell carcinoma or squamous cell carcinoma in situ
g. Squamous cell carcinoma- Bowens Disease, Dysplasia- High Grade, and **Extramammary Paget's***

V. Other applicable diagnoses (*Note: Reasonable efforts should be made to align with above diagnostic options for this organ category prior to the selection of this option.)*

Lymph Node: ~100 Cases

I. Negative for tumor
II. **Positive for tumor***
III. Benign nevus rest
IV. Granulomas
V. Benign or **reactive lymphadenopathy***
VI. Other applicable diagnoses (*Note: Reasonable efforts should be made to align with above diagnostic options for this organ category prior to the selection of this option.)*

Bladder: ~100 Cases

I. **Inverted papilloma***
II. PUNLMP (papillary urothelial neoplasm of low malignant potential)
III. Urothelial carcinoma, invasive
IV. Urothelial carcinoma, non invasive
V. **Carcinoma in situ***
VI. Squamous cell carcinoma
VII. Adenocarcinoma (primary bladder)
VIII. Small cell carcinoma
IX. Metaplasia (squamous, intestinal, nephrogenic)
X. **Inflammatory (Inflammatory pseudotumor, Malakoplakia, Post-operative spindle cell nodule)***
XI. Cystitis cystica/Cystitis cystica glandularis
XII. Papillary cystitis
XIII. Other applicable diagnoses (*Note: Reasonable efforts should be made to align with above diagnostic options for this organ category prior to the selection of this option.)*

2024120583 Wharton et al Supplementary Material

Gynecologic: ~150 Cases

I. Cervix
   a. Squamous cell carcinoma in situ
   b. Invasive squamous cell carcinoma
   c. **Adenocarcinoma in situ***
   d. Invasive adenocarcinoma
   e. High grade squamous intraepithelial lesion
   f. Low grade squamous intraepithelial lesion
   g. **Immature squamous metaplasia***
   h. **Reactive squamous epithelia***
   i. **Reactive glandular changes or squamous metaplasia***
   j. Benign endocervical polyp
   k. Microglandular hyperplasia

II. Endometrium
   a. Proliferative phase endometrium
   b. Secretory phase endometrium
   c. Menstrual phase endometrium
   d. Benign endometrial polyp
   e. Chorionic villi present
   f. Simple endometrial hyperplasia
   g. Atrophic endometrium
   h. **Molar pregnancy (Partial and Complete)***
   i. **Simple or complex endometrial hyperplasia with atypia***
   j. Endometrial adenocarcinoma, grade 1 or 2
   k. Endometrial adenocarcinoma, grade 3
   l. Squamous metaplasia, non-malignant
   m. Villoglandular endometrial adenocarcinoma
   n. Carcinosarcoma
   o. Serous carcinoma
   p. Clear cell carcinoma
   q. Arias-Stella change
   r. Adenosarcoma
   s. Endometritis

III. Ovary, Non-neoplastic
   a. Benign follicle cyst(s)/cystic follicle(s)
   b. Hemorrhagic corpus luteum/luteal cyst
   c. **Polycystic ovarian disease***
   d. Endometriotic cyst/endometriosis
   e. Endosalpingiosis
   f. Ectopic pregnancy

IV. Ovary, Neoplastic, Surface Epithelial
   a. Benign serous cystadenoma/cystadenofibroma
   b. **Borderline serous (papillary) carcinoma***
   c. Mucinous cystadenoma
   d. Invasive serous (papillary) carcinoma
   e. **Borderline/in situ mucinous adenocarcinoma***
   f. Invasive mucinous carcinoma
   g. Endometrioid adenocarcinoma
   h. **Clear cell carcinoma***
   i. Benign Brenner tumor
   j. Malignant Brenner tumor
   k. **Carcinosarcoma***

V. Ovary, neoplastic, sex cord stromal
   a. Granulosa cell tumor
   b. **Sertoli-Leydig cell tumor***
   c. Fibroma/thecoma
   d. **Dysgerminoma***
VI. Ovary, neoplastic, germ cell tumors
   a. Dysgerminoma
   b. Immature teratoma
   c. Carcinoid tumor
   d. Mature (benign) cystic teratoma
   e. Struma
VII. Other applicable diagnoses (*Note: Reasonable efforts should be made to align with above diagnostic options for this organ category prior to the selection of this option.)*

Liver/Bile duct: ~50 Cases

I. **Hepatocellular adenoma***
II. **Nodular regenerative hyperplasia***
III. Focal nodular hyperplasia
IV. **Bile duct adenoma***
V. Bile duct cyst(s)
VI. Hemangioma
VII. **Solitary fibrous tumor***
VIII. **Hepatocyte dysplasia***
IX. **Hepatocellular carcinoma***
X. **Cholangiocarcinoma***
XI. **Lymphoma***
XII. **Chronic hepatitis (viral)***
XIII. **Acute hepatitis***
XIV. **Chronic autoimmune hepatitis***
XV. **Primary biliary cirrhosis***
XVI. **Primary sclerosing cholangitis***
XVII. Steatohepatitis
XVIII. **Metastatic carcinoma***
XIX. Other applicable diagnoses (*Note: Reasonable efforts should be made to align with above diagnostic options for this organ category prior to the selection of this option.)*

Endocrine: ~100 Cases

I. Pancreas
   a. Serous cystadenoma
   b. Mucinous cystic neoplasm
   c. **Intraductal papillary mucinous neoplasm***
   d. Intraductal oncocytic papillary neoplasm
   e. **Ductal adenocarcinoma***
   f. **Pancreatic intraepithelial neoplasia***
   g. Acinar cell cystadenoma
   h. Acinar cell carcinoma
   i. Pancreatoblastoma
   j. Solid pseudopapillary neoplasm
   k. Well differentiated pancreatic endocrine neoplasm
   l. Poorly differentiated pancreatic endocrine carcinoma
   m. **Chronic pancreatitis***
   n. Pseudocyst

II. Thyroid
   a. Multinodular goiter
   b. Colloid nodule
   c. Hashimoto thyroiditis
   d. Fibrosing thyroiditis
   e. Follicular adenoma
   f. Follicular carcinoma
   g. Papillary carcinoma
   h. **Medullary carcinoma***
   i. **Lymphoma***

III. Parathyroid
   a. Adenoma
   b. Hypercellular parathyroid tissue (more than one gland sampled)
   c. **Hypercellular parathyroid tissue (only one sample and no other information)***
   d. Parathyroid carcinoma

IV. Adrenal gland
   a. Adrenal cortical hyperplasia
   b. Adrenal cortical adenoma
   c. **Adrenal cortical carcinoma***
   d. **Pheochromocytoma***
   e. Metastatic carcinoma
   f. Myelolipoma
   g. Adrenal cyst/pseudocyst
   h. No pathological findings

V. Other applicable diagnoses (*Note: Reasonable efforts should be made to align with above diagnostic options for this organ category prior to the selection of this option.)*

Brain/Neuro: ~60 Cases

I. Brain-Neuro: Neoplastic
   a. W.H.O. Grade IV (or 4) tumors [Including: Glioblastoma/Gliosarcoma, **Pineoblastoma***, **Medulloblastoma***, **Primitive neuroectodermal tumor (CNS embryonal tumor)***, **Atypical teratoid rhabdoid tumor***, **Malignant peripheral nerve sheath tumor***]
   b. W.H.O. Grade III (or 3) tumors [Including: **Anaplastic astrocytoma***, **Anaplastic oligodendroglioma***, **Anaplastic ependymoma***, **Choroid plexus carcinoma***, **Anaplastic ganglioglioma***, **Pineal parenchymal tumor of intermediate differentiation***, Primary tumor of the pineal region, **Malignant peripheral nerve sheath tumor***, **Anaplastic meningioma***, **Anaplastic hemangiopericytoma***]
   c. W.H.O. Grade II (or 2) tumors [Including: Pilomyxoid astrocytoma, **Diffuse astrocytoma***, **Pleomorphic xanthoastrocytoma***, **Oligodendroglioma***, **Oligoastrocytoma***, Ependymoma, Atypical choroid plexus papilloma, **Chordoid glioma of the third ventricle***, **Central neurocytoma***, **Extraventricular neurocytoma***,
   d. **Cerebellar liponeurocytoma***, **Pineal parenchymal tumor of intermediate differentiation***, Primary tumor of the pineal region, **Malignant peripheral nerve sheath tumor***, **Atypical meningioma*, Hemangiopericytoma***
   e. W.H.O. Grade I (or 1) tumors [Including: **Subependymal giant cell astrocytoma***, Pilocytic astrocytoma, Subependymoma, Myxopapillary ependymoma, Choroid plexus papilloma, **Angiocentric glioma***, **Gangliocytoma***, **Ganglioglioma***, **Desmoplastic infantile astrocytoma and ganglioglioma***, **Dysembryoplastic neuroepithelial tumor***, Paraganglioma of the spinal cord, **Papillary glanglioneuronal tumor***, **Rosette-forming ganglioneuronal tumor of the fourth ventricle***, **Pineocytoma***, Schwannoma (acoustic neuroma), Neurofibroma, Perineurioma, Meningioma, Hemangioblastoma, Craniopharyngioma, Granular cell tumor of the neurohypophysis, Pituicytoma, **Spindle cell oncocytoma of the adenohypophysis***
   f. **CNS Lymphoma*** (See W.H.O. lymphoma classification)

II. Brain-Neuro: Non-Neoplastic
   a. Any inflammatory disorder (vasculitis, **demyelination***, meningitis, **non-viral encephalitis***, **neurosarcoidosis***)
   b. Any CNS infection **(viral***, parasitic, abscess, etc.)
   c. Arteriovenous malformation
   d. Amyloid angiopathy
   e. **Benign cyst*** (arachnoid, pineal, etc.)
   f. **Cortical dysplasia (seizure disorder)***

III. Other applicable diagnoses (*Note: Reasonable efforts should be made to align with above diagnostic options for this organ category prior to the selection of this option.)*

Kidney, Neoplastic: ~50 Cases

I. Renal cell carcinoma
II. Oncocytoma
III. Angiomyolipoma
IV. **Xanthogranulomatous pyelonephritis***
V. **Inflammatory myofibroblastic pseudotumor***
VI. Urothelial Carcinoma
VII. Other applicable diagnoses (*Note: Reasonable efforts should be made to align with above diagnostic options for this organ category prior to the selection of this option.)*

Salivary Gland: ~50 Cases

I. Pleomorphic Adenoma
II. Warthin Tumor
III. **Oncocytoma***
IV. Mucoepidermoid carcinoma
V. Acinic cell carcinoma
VI. Adenoid cystic carcinoma
VII. Polymorphous low-grade adenocarcinoma
VIII. **Carcinoma ex Pleomorphic Adenoma***
IX. Lymphoma
X. Hemangioma
XI. Lymphoepithelial sialadenitis
XII. **Chronic sclerosing sialadenitis***
XIII. **Necrotizing Sialometaplasia***
XIV. Mucocele
XV. Salivary duct cyst
XVI. Lymphoepithelial cyst
XVII. Other applicable diagnoses (*Note: Reasonable efforts should be made to align with above diagnostic options for this organ category prior to the selection of this option.)*

Hernial/Peritoneal: ~15 Cases

I. Hernia sac
II. Lipoma
III. **Other rare tumors of peritoneal surfaces, such as mesothelioma, solitary fibrous tumor, low grade mucinous tumor, and inflammatory lesions***
IV. Other applicable diagnoses (*Note: Reasonable efforts should be made to align with above diagnostic options for this organ category prior to the selection of this option.)*

Gallbladder: ~15 Cases

I. Chronic cholecystitis
II. Acute cholecystitis
III. Cholesterolosis/Cholesterol polyp

IV. Adenomyoma/adenomatous hyperplasia
V. Mucocele
VI. Adenoma
VII. **High Grade Dysplasia/CIS***
VIII. Invasive adenocarcinoma
IX. Other applicable diagnoses (*Note: Reasonable efforts should be made to align with above diagnostic options for this organ category prior to the selection of this option.)*

Appendix: ~15 Cases

I. No pathologic findings (ie, Normal)
II. Acute appendicitis
III. Mucosal hyperplasia/hyperplastic polyp
IV. Mucocele
V. Adenoma
VI. Mucinous cystadenoma
VII. Low grade appendiceal mucinous neoplasm
VIII. Invasive adenocarcinoma
IX. Fibrous obliteration of lumen
X. Carcinoid tumor
XI. **Goblet cell carcinoid***
XII. Sessile serrated polyp
XIII. Other applicable diagnoses (*Note: Reasonable efforts should be made to align with above diagnostic options for this organ category prior to the selection of this option.)*

Soft Tissue Tumors: ~20 Cases

I. Musculoskeletal—Soft Tissue
  a. Normal
  b. Lipoma
  c. Nodular fasciitis
  d. Myositis ossificans
  e. Localized tenosynovial giant cell tumor (giant cell tumor of tendon sheath)
  f. Diffuse tenosynovial giant cell tumor (pigmented villonodular synovitis)
  g. Benign fibrous histiocytoma
  h. Leiomyoma
  i. Atypical Lipomatous Tumor
  j. **Solitary Fibrous Tumor***
  k. Myxoid liposarcoma
  l. Pleomorphic liposarcoma
  m. **De-Differentiated liposarcoma***
  n. High grade undifferentiated pleomorphic sarcoma
  o. **Synovial Sarcoma***
  p. **Embryonal rhabdomyosarcoma***

II. Musculoskeletal—Bone
  a. Normal
  b. Osteomyelitis
  c. Osteoarthritis
  d. Metastatic carcinoma*
  e. Chondroma
  f. Osteochondroma
  g. Osteoid osteoma
  h. Nonossifying fibroma
  i. Fibrous dysplasia
  j. Bone cyst

k. Aneurysmal bone cyst
l. Chondroblastoma
m. Chondromyxoid Fibroma
n. Grade I chondrosarcoma
o. Grade II chondrosarcoma
p. De-differentiated chondrosarcoma
q. Osteosarcoma (conventional, Intraosseous)
r. Parosteal osteosarcoma
s. High grade pleomorphic sarcoma*
t. Malignant lymphoma*
u. Chordoma

III. Other applicable diagnoses (*Note: Reasonable efforts should be made to align with above diagnostic options for this organ category prior to the selection of this option.)*

Anus/Perianal: ~50 Cases

I. Hypertrophied anal papillae
II. Inflammatory cloacogenic polyp
III. Hidradenoma papilliferum
IV. Condyloma acuminatum
V. Anal canal intraepithelial neoplasia, low grade
VI. Anal canal intraepithelial neoplasia, high grade
VII. Invasive squamous cell carcinoma
VIII. Adenocarcinoma
IX. Melanoma
X. Other applicable diagnoses (Note: Reasonable efforts should be made to align with above diagnostic options for this organ category prior to the selection of this option.)

Supplementary Table S5 Feature list of WSI precision predicate device studies

	Philips 2017		Leica 2019		Hamamatsu 2022		Roche 2024	
**Feature**	**power**	**feature**	**power**	**feature**	**power**	**feature**	**power**	**feature**
small artery	10x	x			10x	x		
psammoma body	10x	x	40x	x	10x	x	40x	x
keratin pearl	10x	x			10x	x		
granuloma	10x	x	20x	x	10x	x	20x	x
adipose cell	10x	x	20x	x	10x	x	20x	x
glandular formation	10x	x			10x	x		
necrosis	10x	x	40x	x	10x	x	40x	x
Reed-Sternberg cell	20x	x	40x	x	20x	x	40x	x
neutrophil	20x	x			20x	x		
plasma cell	20x	x			20x	x		
goblet cell	20x	x	20x	x	20x	x	20x	x
tingible body macrophage	20x	x			20x	x		
foreign body giant cell	20x	x	20x	x	20x	x	20x	x
lymphocyte	20x	x						
nucleolus	40x	x						
eosinophil granules	40x	x			40x	x		
mitosis	40x	x			40x	x		
nuclear membrane	40x	x						
cilia	40x	x			40x	x		
desmosomes	40x	x						
pigment laden macrophages / hemosiderin	40x	x	40x	x	40x	x	40x	x
intraglandular necrosis			20x	x			20x	x
chondrocytes / cartilage			20x	x	10x	x	20x	x
infiltrating or metastatic lobular carcinoma			20x	x	40x	x	20x	x
osteoclasts			20x	x			20x	x
osteocytes			20x	x			20x	x
pleomorphic nucleus of malignant cell			20x	x			20x	x
serrated intestinal epithelium			20x	x			20x	x
skeletal muscle fibers			20x	x	20x	x	20x	x
asteroid bodies			40x	x			40x	x
clear cells			40x	x	20x	x	40x	x
foreign bodies			40x	x			40x	x
megakaryocyte			40x	x			40x	x
osteoid matrix			40x	x	40x	x	40x	x
nuclear grooves			40x	x			40x	x
nerve cell bodies			40x	x			40x	x
duct					10x	x		
nerve					10x	x		
myxoid stroma					20x	x		
calcification					20x	x		
intercellular bridges					40x	x		
intranuclear inclusions					40x	x		
melanin pigment					40x	x		
crystals					40x	x		
**total no. of features**		**21**		**23**		**30**		**23**

References for each study are cited in the main text. Note that the Leica Biosystems GT450 DX study from 2024 used the same feature list as their prior Leica Biosystems 2019 AT2 DX study,[1,2] and the Philips 2024 study (not shown) used the same feature list as the Philips 2017 study.

Abbreviation: WSI, whole slide imaging.

REFERENCES

1. Bauer TW, Behling C, Miller DV, et al. Precise identification of cell and tissue features important for histopathologic diagnosis by a whole slide imaging system. *J Pathol Inform*. 2020;11:3. https://doi.org/10.4103/jpi.jpi_47_19
2. Bauer TW, Hanna MG, Smith KD, et al. A multicenter study to evaluate the analytical precision by pathologists using the Aperio GT 450 DX. *J Pathol Inform*. 2024;15:100401. https://doi.org/10.1016/j.jpi.2024.100401
